# Multi-omics profiling uncovers paradoxical Epstein-Barr virus involvement in autoimmune liver disease pathogenesis

**DOI:** 10.1186/s13568-025-01975-6

**Published:** 2025-10-27

**Authors:** Linyi Zheng, Yuqiang Li, Cenap Güngör, Heming Ge

**Affiliations:** 1 Department of General Surgery, Xiangya Hospital, Central South University, Changsha, 410013 China; 2 Department of General, Visceral and Thoracic Surgery, University Medical Center Hamburg-Eppendorf, Hamburg, Germany

**Keywords:** Autoimmune liver disease, Epstein-Barr virus, Generalized summary-data-based Mendelian randomization, Multi-omics profiling

## Abstract

Autoimmune liver disease (AILD) is a group of immune-mediated chronic liver injury diseases of unknown etiology. The pathogenesis of AILD is not clear, and it poses a serious threat to human life and health for a long time. Several case reports and observational studies have shown that the Epstein-Barr virus (EBV) is directly associated with AILD.However, the causal nature of the intrinsic association remains uncertain.In this study, we investigated the potential association between Epstein-Barr virus and AILDs using a two-sample Mendelian randomization analysis and generalized summary-data-based MR (GSMR) by integrating a genome-wide association study (GWAS) of five antibodies against EBV with the latest GWAS summary statistics for AILDs derived from the FinnGen database. Using summary data-based Mendelian randomization (SMR), we identified AILD-associated candidate genes linked to EBV-related single nucleotide polymorphisms (SNPs). Subsequent transcriptomic and single-cell RNA sequencing analyses were performed to elucidate their functional roles in disease pathogenesis and their interactions with immune cell populations. We found that Epstein-Barr nuclear antigen 1 (EBNA-1) may be a protective factor in autoimmune hepatitis (AIH), while our reverse MR analysis similarly found that autoimmune hepatitis may decrease the levels of EBNA-1 antibodies. More importantly, we found a positive correlation between ZEBRA antibodies and primary biliary cholangitis (PBC). In addition, according to post-GWAS analysis, five genes showed significant causal relationships with AIH and four genes with PBC, of which AIF1-positive B cells may play an important role in AIH, and M1 macrophages may play an important role in PBC progression.Our findings suggest a complex role for EBV in AILD pathogenesis, potentially acting as a double-edged sword. This study represents the first lines of genetic evidence linking EBV and AILDs, opening new avenues for understanding the etiology and potential therapeutic targets in these chronic liver conditions.

**Supplementary Information:**

The online version contains supplementary material available at 10.1186/s13568-025-01975-6.

## Introduction

Autoimmune liver diseases (AILDs) represent a group of chronic progressive inflammatory disorders of the liver and biliary system, characterized by autoimmune mechanisms and often accompanied by the presence of multiple autoantibodies (Trivedi et al. 2024). These diseases are classified based on the primary tissue site of injury, encompassing autoimmune hepatitis (AIH), which predominantly affects hepatocytes; primary biliary cholangitis (PBC); and primary sclerosing cholangitis (PSC), which is characterized by cholangitic injury (Komori 2021). The incidence rates of these diseases vary significantly across populations, with AIH ranging from 0.4 to 2.39 per 100, 000 individuals, PBC from 0.84 to 2.75, and PSC from 0.1 to 4.39 (Trivedi & Hirschfield 2021). Initially considered a chronic inflammatory hepatopathy primarily affecting young Caucasian women, AIH is now recognized to affect individuals of all age groups and ethnicities worldwide. The clinical presentation of AIH is highly variable, ranging from asymptomatic to acute hepatic failure and end-stage liver disease (Czaja 2016).

AILDs progress insidiously, leading to hepatic fibrosis, cirrhosis, and potentially hepatocellular carcinoma (Floreani et al. 2024). Unfortunately, a definitive cure for AILDs remains elusive; the current primary therapy for AIH involves a combination of corticosteroids and azathioprine (Chang et al. 2020). Additionally, ursodeoxycholic acid (UDCA), hormonal agents, and immunosuppressants have demonstrated therapeutic efficacy in patients with PSC and PBC, while endoscopic treatment, such as bile duct dilation and stent implantation, can alleviate the symptoms of PSC. Liver transplantation is the principal therapeutic option for end-stage AILDs, particularly in cases of cirrhosis and hepatic failure, liver transplantation is the principal therapeutic option (Lindor et al. 2022).

Epstein-Barr virus (EBV), a lymphotropic herpesvirus, is a pathogenic agent of infectious mononucleosis (IM) (O’Keefe & Burtson 2023; Rostgaard et al. 2019). EBV was first isolated from cells derived from African Burkitt’s lymphoma, and subsequent research has elucidated its widespread prevalence globally. Previous studies have shown that the pathogenic role of EBV is mediated by a variety of EBV proteins and non-coding RNAs, including the Epstein-Barr nuclear antigen (EBNA) family, latent membrane protein (LMP) family, Epstein-Barr virus-encoded small non-coding RNAs (EBER1 and 2), anti-EB viral capsid antigen (VCA) family, anti-EBV diffuse early antigen (EA-D), and transcriptional transactivator proteins such as ZEBRA (Huang et al. 2022).

EBV has been epidemiologically linked to the development of various autoimmune diseases including systemic lupus erythematosus, Sjögren’s syndrome, rheumatoid arthritis, and multiple sclerosis (Bjornevik et al. 2023; Laderach et al. 2025; Robinson et al. 2024), which is associated with EBV possibly causing tissue damage by activating the immune system (Morawiec et al. 2025). However, there are limited studies on the relationship between EBV infection and AILDs, with existing research consisting mainly of observational studies and case reports. Some case reports have suggested that EBV may trigger AIH, and some studies have found an association between PBC and EBV infection (Wada et al. 2014). Nevertheless, comprehensive mechanistic studies linking EBV infection with the development of AILDs are lacking (Chiba et al. 2004).

**Fig. 1:**
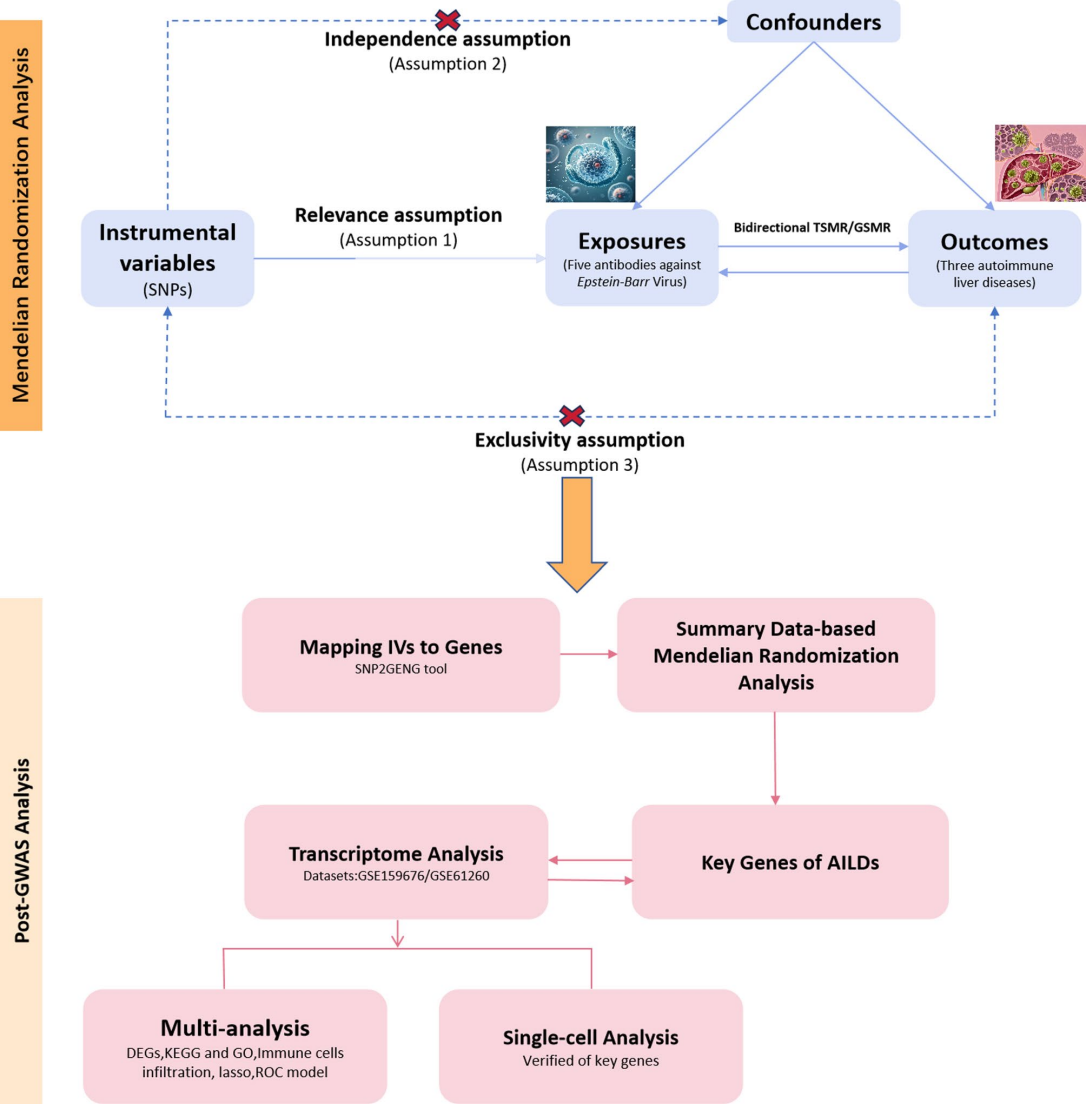
Schematic diagram of this study

In this study, we aimed to determine the causal link between EBV infection and AILDs. To achieve this, we employed Mendelian randomization (MR), a robust analytical framework that utilizes common genetic variants as instrumental variables (IVs), to explore causal associations between exposures and outcomes. MR leverages the random allocation of genotypes at conception, thereby mitigating confounding biases prevalent in conventional epidemiological studies (Davies et al. 2018). By mimicking the randomization process inherent in clinical trials, MR enhances the robustness of causal inference (Cornish et al. 2019). Conventional Mendelian randomization approaches, encompassing inverse variance weighted (IVW), weighted median, and MR-Egger estimators, were augmented with sensitivity analyses. We further implemented generalized summary-data-based MR (GSMR), a method enhancing causal inference efficiency by explicitly modelling linkage disequilibrium (LD) architecture.In this framework, genome-wide significant variants associated with pathogen-specific antibody titers serve as instrumental variables to delineate causal effects on clinical endpoints. By circumventing confounding inherent in observational designs, GSMR provides robust causal estimates of infectious agents in complex disease etiology.Applied to EBV and AILDs, this integrated approach established robust causal inference between serological responses and disease pathogenesis. Complementarily, transcriptomic profiling of autoimmune hepatitis (AIH) and primary biliary cholangitis (PBC) cohorts revealed shared diagnostic genes and interacting molecular pathways, revealing EBV-associated immune perturbations underlying disease convergence (Fig. 1).

## Materials and methods

### Data sources for AILDs and EBV

Summary statistics for Epstein-Barr virus (EBV) serological traits, including anti-EBV IgG seropositivity and antibodies against EA-D, EBNA-1, VCA p18, and ZEBRA were sourced from genome-wide association studies (GWAS) reported by Butler-Laporte et al. (Butler-Laporte et al. 2020). These datasets encompassed 8, 735 to 8, 518 individuals (Table 1) and were publicly accessible via the MRC-IEU UK Biobank OpenGWAS repository (IDs: ebi-a-GCST90006897 to ebi-a-GCST90006901). For AILDs, we utilized the updated FinnGen GWAS data (November 2024 release). Case–control cohorts comprised:AIH: 264 cases / 372, 273 controls, PBC: 760 cases / 372, 273 controls, and PSC: 2, 317 cases / 437, 418 controls (European ancestry). All GWAS data for exposures and outcomes were derived from European-ancestry populations to minimize population stratification bias.

**Table 1:** Detailed information on studies and datasets used for Mendelian randomization analysis

Exposure/Outcome	Data source	Phenotype	Population	Sample size
Autoimmune liver diseases (AILDs)	The FinnGen consortium	Autoimmune hepatitis	European	264/372273
		Primary biliary cholangitis	European	760/372273
		Primary sclerosing cholangitis	European	2317/437418
Antibodies against Epstein-Barr Virus	Butler-Laporte et al. ^16^	IgG	European	8735
		EA-D	European	7763
		EBNA-1	European	7972
		VCA-p18	European	8518
		ZEBRA	European	8191

### SNPs selection

In order to guarantee the precision and validity of the causal relationship between antibodies against EBV and AILDs, the following restrictions were added to the IV inclusion criteria.Firstly, in the selection of IV, we initially strictly used snp with a standard *p* value of less than 5*10^−8^ as the inclusion criteria, but after using this filtering method, too little snp was obtained to perform normal MR analysis, so after multiple debugging, we determined that only snp with *p* < 5*10^−6^ was included as IV. This inclusion criterion has been applied in several authoritative Mendelian randomization-related studies, including a Mendelian randomization study published in Nature Genetics by Sanna et al. in 2019 (Sanna et al. 2019). Results from analyses performed at the 5*10^−8^ threshold are placed in Table S13 as exploratory results.To explore the causality of the reverse association, the following selection criteria were employed for genetic instruments: a set of SNPs meeting the GWAS significance threshold (*p* < 5*10^−8^), which were associated with AILDs characteristics. However, for Primary Biliary Cholangitis and Autoimmune Hepatitis, the GWAS significance threshold was adjusted to *p* < 5*10^−6^ due to an insufficient number of SNPs (< 3), which precluded proper program execution.Second, quality control removed SNPs exhibiting linkage disequilibrium (LD; r^2^ < 0.001) within 10, 000 kb windows using TwoSampleMR. Palindromic SNPs were excluded to ensure consistent directionality of the allele effects. Instrument strength was quantified using F-statistics, retaining variants with F > 10 and a minor allele frequency (MAF) > 0.01 (Hemani et al. 2018). To satisfy the MR exclusion criteria, the LDtrait was screened for pleiotropic SNPs associated with autoimmune liver disease traits. (Tables S7–S9).

### Statistical analysis

The GSMR approach was used in the primary analysis. This methodology is a technically sophisticated technique that conducts MR analyses using multiple instruments with a high degree of independence to ascertain the causal relationships between risk factors and diseases. This is achieved by leveraging data at the summary level from GWAS studies that are independent.In reverse analyses, for the GWAS summary statistics of primary Sclerosing Cholangitis, we selected independent SNPs by setting an LD threshold of r2 < 0.05 and a *P*-value threshold of 5*10^−8^. Conversely, for the GWAS of Primary biliary cholangitis and autoimmune hepatitis, we applied a genome-wide *P*-value threshold of 5*10^−6^ because of the limited number of SNPs (Tables S5 and S6). In addition, the results of all analyses performed at the 5*10^−8^ threshold are presented in Supplementary Table S13 (Table S13). Subsequently, we applied the HEIDI-outlier approach to eliminate instruments exhibiting strong putative pleiotropic effects, specifying a *p*-value threshold of 0.01 for HEIDI-outlier filtering analysis.

To further support our view, in addition to using the GSMR method, the “Two-Sample MR” package was utilized to execute the formatting, harmonization, and semi-automated analysis of genetic association study summary data. The statistical significance of the results was determined using a *p*-value cutoff of 0.05. Following the selection of valid SNPs, we augmented our Mendelian randomization analysis by applying five additional MR methods to all antibody-disease relationships from the main analysis. These supplementary methods include the IVW method, MR-Egger regression, and weighted median approaches (Chen et al. 2022a, b).

### Criteria for assessing significance of results

To address multiple testing, a Bonferroni-corrected significance threshold was set at *p* < 0.05 / 30 = 1.67*10^−3^ to account for multiple testing across the five EBV antibodies and six AILD outcomes. Significance required concordant directional estimates across the four methods (GSMR, IVW, MR-Egger, and weighted median). The GSMR surpassed this threshold, with no evidence of pleiotropy detected via MR-PRESSO global tests or modified Q-statistics. Analyses were conducted in R 4.3.0, using TwoSampleMR and gsmr2 packages.

## Summary data-based Mendelian randomization (SMR) analysis

To elucidate molecular mechanisms underlying the causal relationships between EBV infection and AILDs, lead SNPs serving as IVs were analyzed through the FUMA GWAS platform (v1.3.8)—an integrative framework for GWAS result annotation, functional mapping, and visualization (Watanabe et al. 2017). To functionally link these genomic loci with EBV infection/AILDs pathogenesis, we conducted transcriptome-wide Mendelian randomization analyses of whole-blood expression quantitative trait loci (eQTLs) employing the SMR method (Zhu et al. 2016). This approach demonstrates enhanced statistical power over conventional MR when interrogating gene expression-trait associations, particularly for polygenic phenotypes. Cis-eQTL (cis-expression quantitative trait loci) data for genes were sourced from the eQTLGen consortium (https://eqtlgen.org/) (Vosa et al. 2021). Analyses were conducted using the SMR framework (v1.3.1) with integrated heterogeneity in dependent instruments (HEIDI) testing, strictly adhering to pre-specified analytical parameters. The HEIDI procedure enabled the differentiation of vertical pleiotropy from causal associations, with a threshold of *p*-HEIDI > 0.05, establishing evidence against alternative causal pathways (Dai & Feng 2025).

### Colocalization analysis

To investigate whether the genetic associations for AILDs might be mediated by effects on gene expression, we performed colocalization analyses using the Bayesian framework implemented in the R package ‘coloc’. This analysis tests whether the GWAS and for gene expression (expression quantitative trait loci, eQTLs) within a given genomic region are likely to be driven by the same underlying causal variant (Liu et al. 2025).

For each AILD-risk locus and its corresponding cis-eQTL, we extracted all common SNPs within a ± 1 Mb window centred on the lead cis-eQTL variant. We then evaluated five mutually exclusive hypotheses:H0: No causal variant for either trait.H1: A causal variant for gene expression only.H2: A causal variant for disease risk only.H3: Two distinct, nearby causal variants for each trait.H4: A single shared causal variant for both traits.A posterior probability for H4 (PPH4) > 0.6 was set as the threshold for strong evidence of colocalization. A significant colocalization result suggests that the GWAS locus influences disease risk by modulating the expression level of the target gene (Chen et al. 2022a, b).

### Gene set enrichment analysis

Protein-level gene interactions were analyzed using Gene Ontology (GO) and Kyoto Encyclopedia of Genes and Genomes (KEGG) enrichment analyses using ClusterProfiler. Species-specific annotations derived from org.Hs.eg.db defined gene identities (*p* < 0.05). Publication-ready visualizations were generated using ggplot2 and an enrichment plot, ensuring analytical reproducibility.

### GEO data sources and differential gene analysis

The NCBI public database of gene expression provided autoimmune hepatitis data from which dataset GSE159676 was selected for subsequent analysis, including 6control cases and 3 disease cases, while the PBC-related transcriptome data file was GSE61260, including 38 control cases and 11 disease cases, using R software (version 4.4.1) and the limma package. We performed downloaded boxplots and principal component analysis (PCA) on the dataset to verify the data and *p*-value DEGs, applying the selection criteria of | log2 (fold change) |> 0.5 and identify < 0.05. As each dataset (GSE159676 and GSE61260) was processed in a single batch, no inter-batch normalization was required.Heatmaps andvolcano plotswere created using the R packages “heatmap” and “ggplot2, “ respectively.For the purpose of identifying and selecting significant disease diagnostic markers, the “glmnet” R package was employed to apply LASSO logistic regression.The LASSO regression was performed with tenfold cross-validation, and the optimal penalty parameter (lambda) was selected based on the criterion of lambda.min.The response variable for the LASSO regression was “PBC disease status”.In addition to validate differentially expressed genes (DEGs), we have performed an independent validation using the GEO dataset GSE304352.

### Analysis of immune cell infiltration

Immune cell infiltration profiles in the liver tissue were deconvoluted using the CIBERSORT algorithm to quantify the relative abundances of 22 distinct immune cell subsets. Additionally, the xCell algorithm was used to quantify immune infiltrates in AIH and PBC microenvironments and to calculate immune and mechanistic proportions for each patient.Subsequently, Spearman’s rank correlation analysis have shown associations between tissue-specific gene expression signatures and immune cell composition (Newman et al. 2015).

#### Single-cell data processing and analysis

Single-cell RNA sequencing data of PBMC from patients with AIH (*n* = 4) and healthy controls (*n* = 4) (GSE186333) were processed using Seurat (v4.2.0). Stringent quality control thresholds were applied: cells with 200–10, 000 unique molecular identifiers (nFeature_RNA), mitochondrial gene content < 20% (percentage mt), and total RNA counts < 15, 000 (nCount_RNA) were retained, and normalized data were subjected to PCA, followed by harmony integration for batch correction. Cellular clusters were visualized via t-distributed stochastic neighbor embedding (t-SNE), a nonlinear dimensionality reduction technique optimized for high-dimensional data projection. Cell identities were annotated through systematic curation of published marker genes, leveraging CellMarker and PanglaoDB databases (Butler et al. 2018). Subtype-specific marker genes were defined by an absolute average log2-fold change > 0.585 and an adjusted *p*-value < 0.05, enabling precise immune subset characterization (Osorio et al. 2022).

#### Trajectory analysis

Pseudotime analysis was performed using Monocle3 to reconstruct cellular developmental trajectories based on gene expression dynamics. The algorithm constructed cell datasets, identified highly variable genes across clusters, applied dimensionality reduction, and generated tree-like structures representing pseudotemporal ordering (Zhao et al. 2024).

## Result

### Exploration of the causal effect of EBV infection on AILDs

#### The causal effect of anti–EBV EA-D antibody on AILDs

**Fig. 2:**
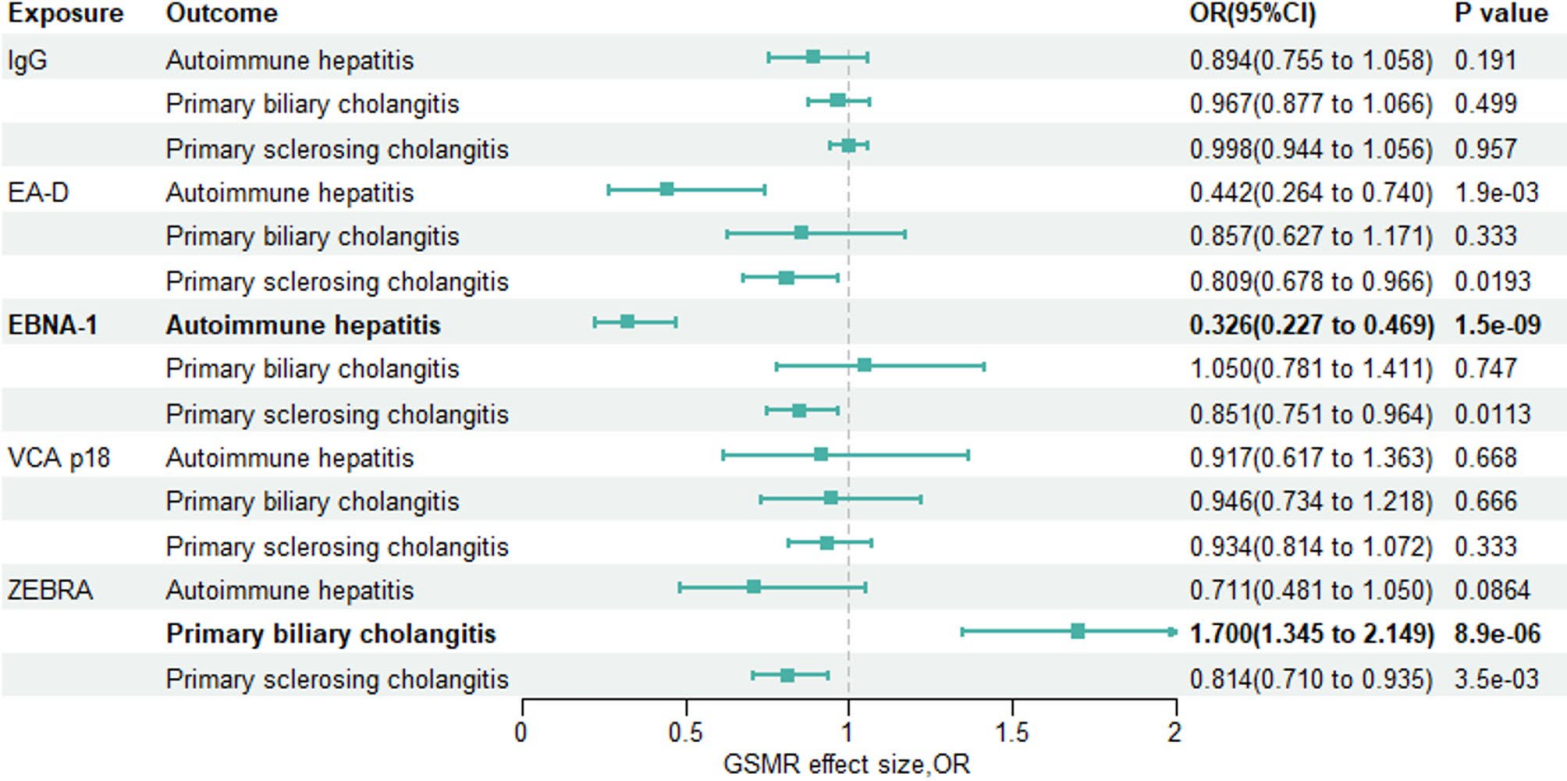
Forest plot for the GSMR effect of antibodies against EBV on AILDs. For GSMR analyses, a Bonferronicorrected threshold of *P* = 1.67*10^−3^ was considered significant

Initially, we probed the causal impact of five Epstein-Barr virus antibodies on three autoimmune liver diseases via GSMR and two-sample MR analyses (Tables S1–S2, Fig. 2 and Figs. S1–S6, S13–S18). Power calculations via mRnd indicated 27% at *p* < 5*10⁻^8^ vs. 60% at *p* < 5*10⁻^6^, the above conclusions further demonstrate the reliability of our choice of this threshold as a screening condition. GSMR analysis revealed a negative association between genetically elevated anti-EBV EA-D antibody levels and both autoimmune hepatitis (OR: 0.44, 95% CI: 0.26 to 0.74, *p* = 0.0019) and primary sclerosing cholangitis (OR: 0.81, 95% CI: 0.68 to 0.97, *p* = 0.019).

#### The causal effect of anti–EBV EBNA-1 antibody on AILDs

Subsequent analysis revealed an association between anti-EBV EBNA-1 antibody and autoimmune liver diseases (AILDs). The GSMR results indicated a negative correlation between anti-EBV EBNA-1 antibodies and primary sclerosing cholangitis (OR, 0.85; 95% CI: 0.75 to 0.96; *p* = 0.011). Notably, we observed a strong negative correlation between anti-EBV EBNA-1 antibody and autoimmune hepatitis (OR: 0.33, 95% CI: 0.23 to 0.47, *p* = 1.49*10^−9^).

#### The causal effect of anti–EBV ZEBRA antibody on AILDs

In a subsequent study, we examined the association between anti-EBV ZEBRA antibodies and AILDs. The GSMR results highlighted a negative correlation between anti-EBV ZEBRA antibody and primary sclerosing cholangitis (OR, 0.81; 95% CI: 0.71 to 0.93; *p* = 0.0034). Notably, we uncovered a robust positive correlation between anti-EBV ZEBRA antibodies and primary biliary cholangitis (odds ratio [OR]: 1.70, 95% CI: 1.35 to 2.15, *p* = 8.88*10^−6^).

### The causal effect of AILDs on EBV infection

#### The causal effect of AILDs on anti–EBV EA-D antibody

**Fig. 3:**
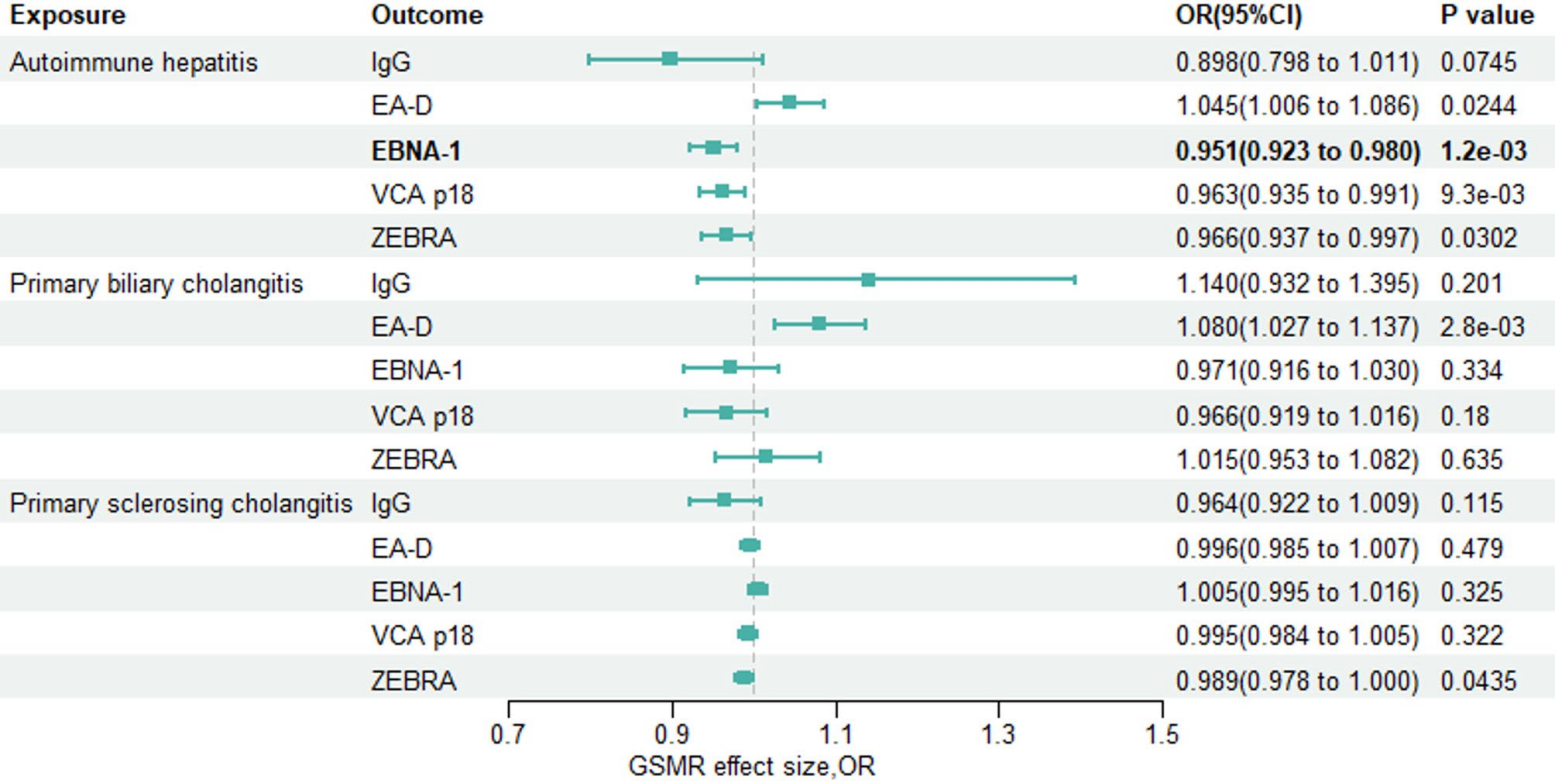
Forest plot for the GSMR effect of AILDs on antibodies against EBV. For GSMR analyses, a Bonferronicorrected threshold of *P* = 1.67*10^−3^ was considered significant

To further explore the causal effect of AILDs on anti-EBV antibodies, we performed MR analyses to test the existence of the reverse or bidirectional causal relationships between AILDs and EBV infection (Tables S3–S4, Fig. 3 and Figs. S7–S12, S19–S24). We found a positive association between primary biliary cholangitis and increased anti-EBV EA-D antibody levels (OR: 1.08, 95% CI: 1.03 to 1.14, *p* = 0.0016) as well as between autoimmune hepatitis and anti-EBV EA-D antibody levels (OR: 1.05, 95% CI: 1.01 to 1.09, *p* = 0.024).

#### The causal effect of AILDs on anti–EBV EBNA-1 antibody

Subsequent analysis revealed the effect of AILDs on anti-EBV EBNA-1 antibodies, and we observed a strong negative correlation between autoimmune hepatitis and anti-EBV EBNA-1 antibodies (OR: 0.95, 95% CI: 0.92 to 0.98, *p* = 1.2*10^−3^).

#### The causal effect of AILDs on anti–EBV VCA p18 antibody

Next, we investigated the relationship between genetically driven autoimmune hepatitis and anti-EBV VCA p18 antibody levels. The analysis demonstrated a significant negative association (OR: 0.96, 95% CI: 0.94–0.99, *p* = 0.0093), indicating that genetic factors contributing to autoimmune hepatitis may be protective against increased levels of anti-EBV VCA p18 antibodies.

#### The causal effect of AILDs on anti–EBV ZEBRA antibody

Furthermore, we examined the impact of genetic predictors of autoimmune hepatitis on the anti-EBV ZEBRA antibody levels. Our results showed a negative effect, albeit with a wider confidence interval (OR: 0.97, 95% CI: 0.94–1.00, *p* = 0.03), suggesting a trend towards lower anti-EBV ZEBRA antibody levels in individuals with a genetic predisposition to autoimmune hepatitis.

### Post-GWAS analysis of EBNA-1 associated SNPs on AIH

#### Functional enrichment analysis and SMR analysis of EBNA-1 associated genes on AIH

Further analyses were conducted to investigate the biological significance of expression quantitative trait loci (eQTL) genes, demonstrating causal relationships with EBV infection and their potential involvement in AILDs. SNP-based gene mapping of instrumental variables for anti-EBV EBNA1 antibody titers performed using the SNP2GENE platform identified 15 proximal genes showing strong cis-regulatory associations (Table S9).

**Fig. 4:**
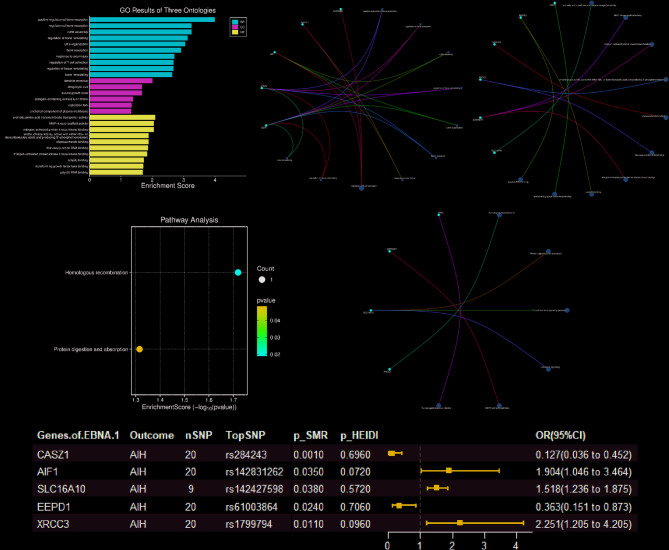
Functional enrichment analysis and SMR analysis of EBNA-1 associated genes on AIH. **A** GO functional enrichments of three Ontologies (BP, CC and MF). **B** Significant GO functional enrichments based on Biological Process (BP). **C** Significant GO functional enrichments (FDR *p-value* < *0.05*) based on Molecular Function (MF). **D**, **E** KEGG pathway enrichment analysis of genes mapping to EBNA-1. **F** Forest plot of SMR analysis of the causal effect of EBNA-1-related genes on significant AIH

Functional annotation revealed distinct pathway enrichments through GO analysis, with significant associations with biological processes, including positive regulation of bone resorption, ruffle assembly, and molecular functions such as MAP-kinase scaffold activity and aromatic amino acid transmembrane transport (Fig. 4A-C). KEGG pathway analysis further implicated homologous recombination and protein digestion/absorption mechanisms (Fig. 4D, E).

Following rigorous statistical controls using HEIDI-outlier testing (*p* < 0.05) and SMR analysis (*p* > 0.05), five genes maintained causal associations with AILD pathogenesis: CASZ1, AIF1, SLC16A10, EEPD1, and XRCC3 (Fig. 4F). Co-localization results for these representative genes are shown in Supplementary Table S14 (Table S14), where co-localization results for AIF1 (PPH4 = 0.998) with CASZ1 (PPH4 = 0.78) show strong evidence supporting co-localization of GWAS and eQTL signals.

#### Identification of differentially expressed genes and immune infiltration

**Fig. 5:**
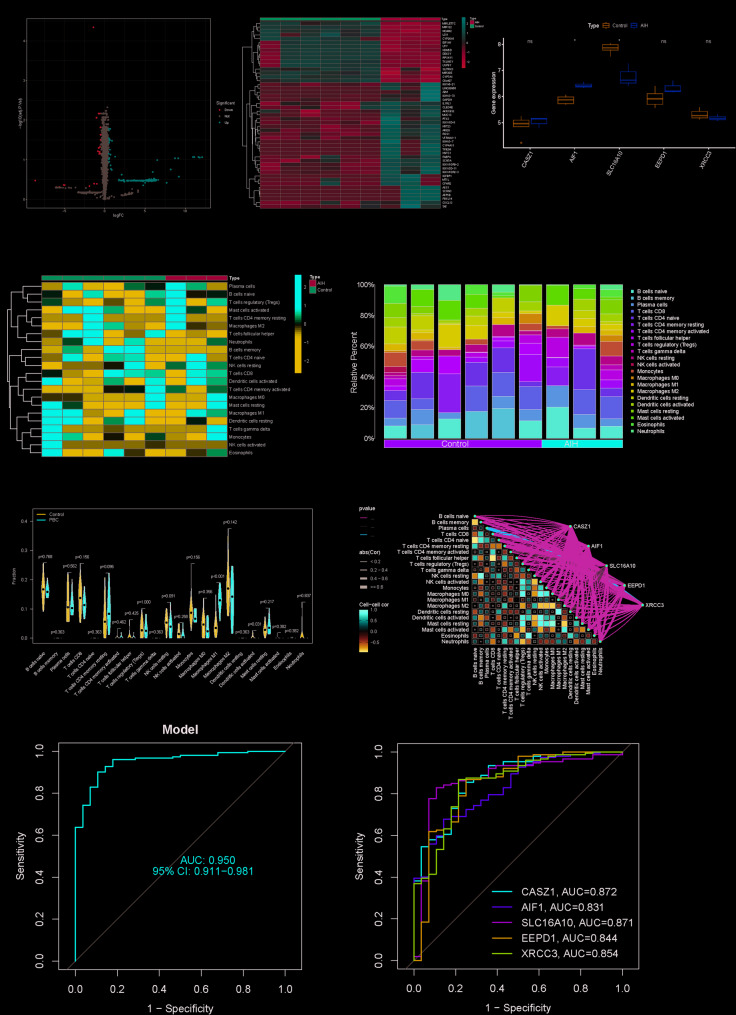
Identification of differentially expressed genes and immune infiltration. **A** Volcano plot of gene expression profiles according to data from the GSE159676 dataset. **B** Heatmap of the AIH-related DEGs with the greatest fold changes in expression. **C** Expression levels of CASZ1, AIF1, SLC16A10, EEPD1, and XRCC3 in the control and AIH groups from the GSE159676 dataset. **D** Heatmap of the intercorrelations among the 22 immune cell types. **E** Stacked bar chart illustrating the distribution of immune cell populations in AIH and control samples. **F** Violin plot comparing the distribution and relative abundance of 22 immune cell subsets between control and AIH groups. **G** The association between five key genes and 22 immune cell subsets. (The different types of lines represented the positive or negative relations; The different colors of the lines represented the *p-values* of the relations, and the thickness of the lines represented the strength of the relations.Using Spearman’s rank correlation method.) **H** ROC curve for the combined dataset of five genes. **I** ROC curves for each of the five related genes

To validate these findings, we analyzed the GSE159676 dataset containing transcriptomic profiles from 3 AIH specimens and 6 normal controls.Comparative analysis identified 49 differentially expressed genes (DEGs) in AIH tissues (16 downregulated, 33 upregulated; fold-change > 0.5, *p* < 0.05) (Fig. 5A, B, Table S11). Notably, our results demonstrated significantly elevated expression of AIF1 in patients with AIH compared to controls, while the expression of SLC16A10 showed the opposite trend (*p* < 0.05). In contrast, CASZ1, EEPD1, and XRCC3 showed no statistically significant differences between the groups (Fig. 5C). Notably, key differentially expressed genes identified in our primary analysis, including AIF1 and SLC16A10, were successfully replicated in the independent validation dataset (GSE304352). (Table S16).

Subsequent analyses characterized the landscape of immune infiltration and intercellular correlations within the hepatic microenvironment. We observed heterogeneous immune cell engagement and intricate network-level interactions across the cohorts. Quantitative profiling of the 22 immune cell subtypes revealed significant compositional shifts in AIH specimens relative to controls (Fig. 5D, E), with B-lymphocyte populations demonstrating marked enrichment in AIH microenvironments (Fig. 5F, Table S17), while xCell immunoinfiltration analysis showed significant enrichment of CD8 + T cells with iDCs in the AIH group (Fig. S27, Table S19). In order to further explore the relationship between the above five AIH highly related genes and immune cells, we also performed correlation analysis between the above five genes and 22 immune cells, and found a strong negative correlation between SLC16A10 and Macrophages M1, EEPD1 and CD4 activated memory T cells, and SLC16A10 and Dendritic resting cells, while there was a strong positive correlation between XRCC3 and Plasma cells (Fig. 5G). To assess diagnostic potential, we performed receiver operating characteristic (ROC) analysis, revealing a combined area under the curve (AUC) of 0.950 for the five-gene panel (Fig. 5H, I).

#### Analysis of EBNA-1 associated genes with AIH single cells

**Fig. 6:**
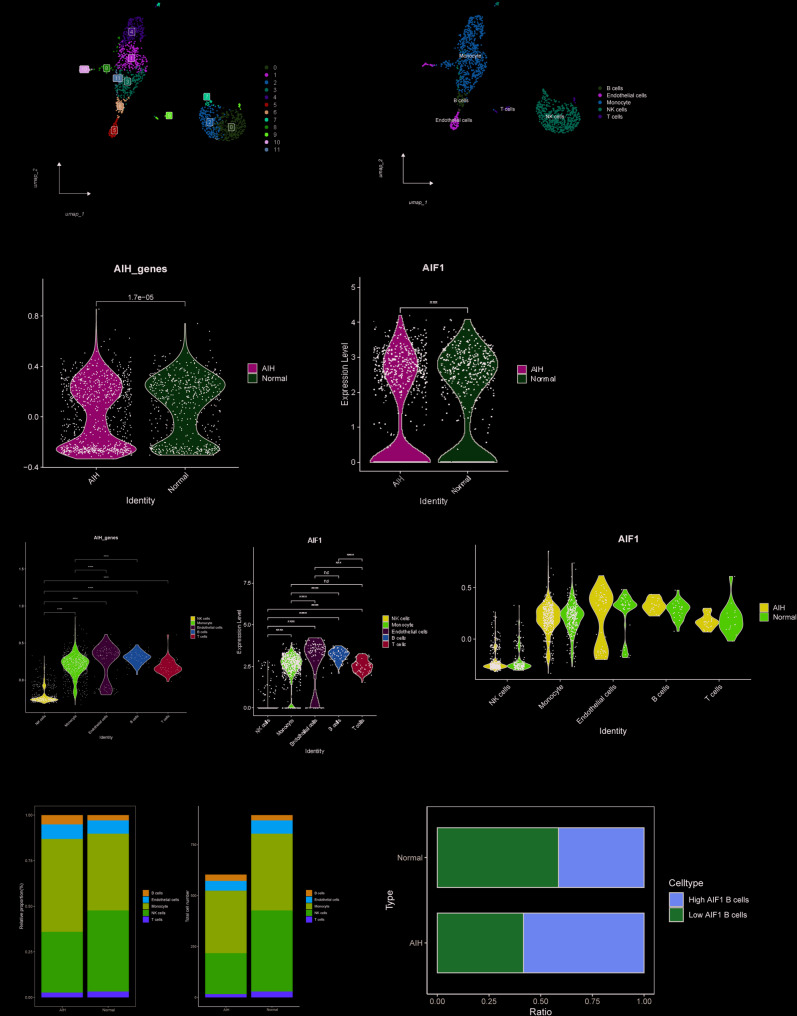
The single-cell transcriptomic analysis of PBMCs in AIH patients. **A** UMAP plot illustrating the distribution of 12 identified cell populations. **B** Split UMAP plots showcasing the distribution of the 14 cell populations within PBMCs. **C** Violin plot showing the distribution differences of AIH-related genes in different samples. **D** Violin plot showing the distribution differences of AIF1 in different samples. **E** Violin plot showing the distribution differences of AIH-related genes in five immune cells. **F** Violin plot showing the distribution differences of AIF1 in five immune cells. **G** Violin plots showed differences in the distribution of AIF1 among the five immune cells in AIH and control samples. **H** Stacked bar chart illustrating the distribution of five immune cell populations in AIH and control samples. **I** Stacked bar chart illustrating the different expression of AIF1 in B cells of different samples

We performed single-cell RNA sequencing of peripheral blood mononuclear cells (PBMCs) from autoimmune hepatitis patients and healthy controls. Following initial quality control to exclude low-quality cells and genes, we applied principal component analysis to reduce data dimensionality. The Harmony algorithm was employed to correct for batch effects, effectively integrating samples across batches into a unified data space and minimizing technical variability (Fig. S25A–J). After the above series of steps, we identified five distinct cell populations (Fig. 6A, B). Comparative expression analysis of five conserved diagnostic genes (CASZ1, AIF1, SLC16A10, EEPD1, and XRCC3) revealed significantly elevated expression levels in patients with AIH compared to healthy individuals (Fig. 6C). Notably, AIF1 expression showed the most pronounced upregulation in patients with AIH, suggesting its potential utility as a diagnostic biomarker for AIH (Fig. 6D). AUCell scoring analysis demonstrated that these five genes, particularly AIF1, were predominantly expressed in endothelial and B cells, with significantly higher expression levels in patients compared than in controls (Fig. 6E, F). Furthermore, comparative analysis of cellular composition revealed a marked increase in the proportion of B cells in AIH patients relative to healthy controls (Fig. 6G, H). Importantly, we observed a significant enrichment of AIF1-positive B cells in the AIH cohort, highlighting the potential role of this cell population in disease pathogenesis (Fig. 6I). To characterize the dynamic changes in gene expression patterns between AIF1 + B cells and AIF-B cells, differentiation trajectories were constructed using Monocle3. Results are placed in Supplementary Figure S26 (Fig. S26A–E).

### Post-GWAS analysis of ZEBRA associated SNPs on PBC

#### Functional enrichment analysis and SMR analysis of ZEBRA associated genes on PBC

**Fig. 7:**
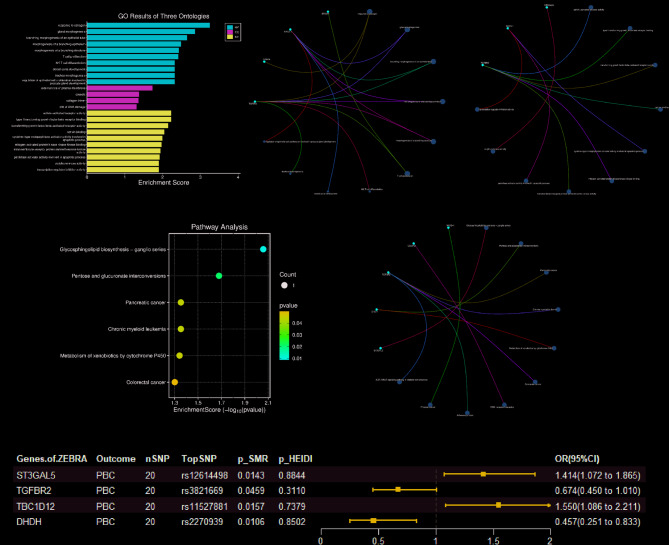
Functional enrichment analysis and SMR analysis of EBNA-1 associated genes on PBC. **A** GO functional enrichments of three Ontologies (BP, CC and MF). **B** Significant GO functional enrichments based on Biological Process (BP). **C** Significant GO functional enrichments (FDR *p-value* < *0.05*) based on Molecular Function (MF). **D**, **E** KEGG pathway enrichment analysis of genes mapping to ZEBRA. **F** Forest plot of SMR analysis of the causal effect of ZEBRA-related genes on significant PBC

Similarly, we performed gene mapping of IVs of the anti-EBV ZEBRA antibody and identified 10 closely related genes (Table S10). Functional annotation of these candidates revealed a striking enrichment in estrogen-responsive pathways and developmental processes, including epithelial tube morphogenesis. Molecular characterization highlighted TGF-β superfamily interactions through type I and activin-activated receptor binding (Fig. 7A–C). KEGG pathway analysis further implicated the glycosphingolipid biosynthesis and carbohydrate metabolism networks (Fig. 7D, E). Application of HEIDI-outlier filtering (*p* > 0.05) with SMR analysis (*p* < 0.05) refined four causal genes (ST3GAL5, TGFBR2, TBC1D12, and DHDH), demonstrating pleiotropic associations with PBC susceptibility (Fig. 7F). Co-localization results for these representative genes are shown in Supplementary Table S15 (Table S15),

#### Identification of differentially expressed genes and immune infiltration

**Fig. 8:**
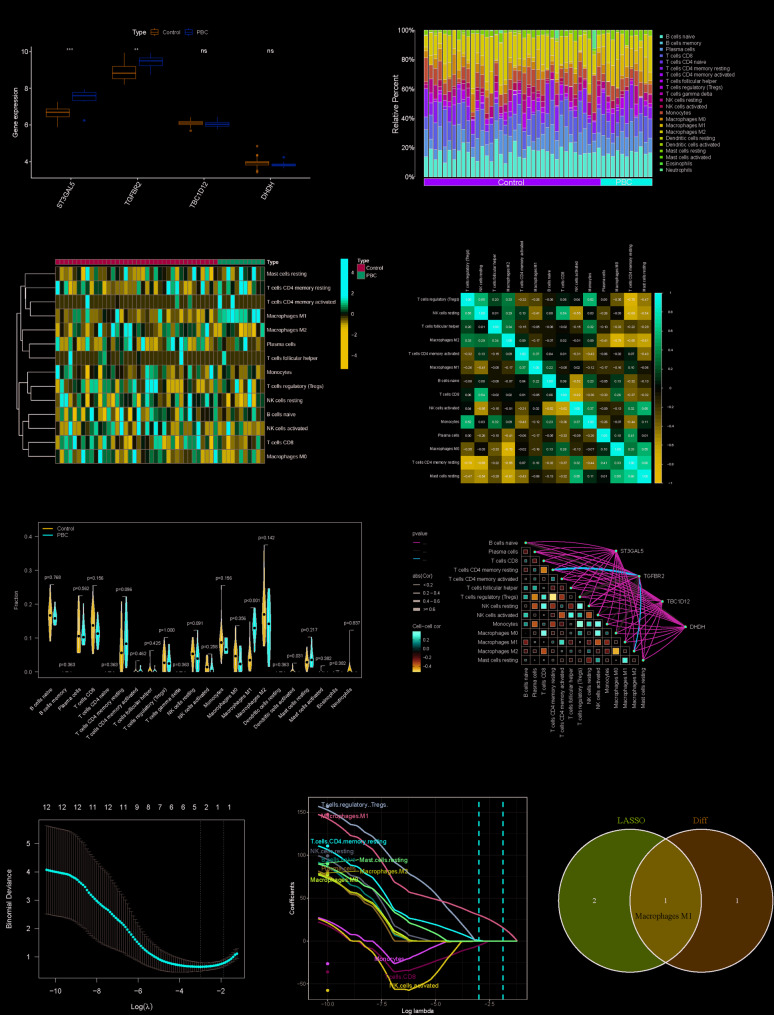
Identification of differentially expressed genes and immune infiltration. **A** Expression levels of ST3GAL5, TGFBR2, TBC1D12 and DHDH in the control and PBC groups from the GSE61260 dataset. **B** Stacked bar chart illustrating the distribution of immune cell populations in PBC and control samples. **C** Heatmap of the intercorrelations among the 22 immune cell types. **D** Pearson correlations among 22 immune cells, negative in blue and positive in red. **E** Violin plot comparing the distribution and relative abundance of 22 immune cell subsets between control and PBC groups. **F** The association between five key genes and 22 immune cell subsets. **G** LASSO regression optimally selected the most relevant immune cell types. **H** Confidence interval of each Lambda of LASSO regression. **I** Venn diagram showing core immune cells in PBC

Using the GSE61260 dataset, we conducted a comprehensive analysis of the four candidate genes in both the control and disease cohorts. Our results demonstrated significantly elevated expression of ST3GAL5 and TGFBR2 in patients with PBC compared to controls (*p* < 0.05). In contrast, TBC1D12 and DHDH levels exhibited no statistically significant differences between the groups (Fig. 8A, Table S12). To assess the diagnostic potential, we performed ROC analysis, which revealed a combined area under the curve (AUC) of 0.876 for the four-gene panel. Individually, ST3GAL5 (AUC = 0.800) and TGFBR2 (AUC = 0.805) showed modest predictive capacity (Fig. S29).

We next evaluated 22 immune cell infiltration patterns in PBC versus controls, uncovering distinct compositional shifts (Fig. 8B–D). Notably, M1 macrophages (*p* < 0.001) and activated dendritic cells (*p* = 0.03) were significantly enriched in the PBC samples (Fig. 8E). Among them, significant differences in activated dendritic cells were also validated in our xCell immune infiltration algorithm performed (Fig. S28, Table S20). Correlation analysis between the four candidate genes and 22 immune cell subtypes revealed that TGFBR2 was negatively associated with M0 macrophages, activated NK cells, monocytes, Tregs, and CD8 + T cells but positively correlated with resting CD4 + memory T cells and naïve B cells (Fig. 8F, Table S18). To explore which class of immune cells may contribute to PBC, we analyzed the above 22 immune cells using LASSO regression and integrated them with differential immune cell results from previous analyses, which showed that M1 Macrophages suggests a potential role for PBC (Fig. 8G–I). These findings suggest a potential interplay between dysregulated gene expression and immune microenvironment remodeling in PBC pathogenesis.

## Discussion

As a complex disease influenced by both genetic and environmental factors, the association between AILDs and viral infections, particularly EBV, remains unclear. To address this challenge, bidirectional MR analysis was conducted to illuminate the causal relationships between EBV infection and AILDs. MR findings highlighted that genetically predicted anti-EBV antibodies were causally implicated in AILD pathogenesis. Specifically, anti-EBV EBNA-1 antibodies were associated with a reduced risk of AIH, whereas anti-EBV ZEBRA antibodies were correlated with an elevated risk of PBC. Transcriptomic profiling identified AIF1 and four other genes as potential diagnostic biomarkers linking EBNA-1 seropositivity to AIH. Furthermore, AIF1-positive B cells were enriched in patients with AIH, suggesting a possible role in disease progression. Similarly, ST3GAL5 and three co-regulated genes emerged as candidate biomarkers connecting ZEBRA immunity to PBC, with M1 macrophages potentially mediating ZEBRA-driven pathogenic effects. This suggests a mechanistic interplay between Epstein-Barr virus exposure and the development of AILDs, highlighting distinct antigen-specific immune responses in AIH and PBC pathogenesis.

EBV deems among the most prevalent of all human viruses and a well-known trigger of autoimmune diseases.On the other hand, the “hygiene hypothesis” suggests that different microbes in the environment may have immunomodulatory effects and exposure to bacterial or viral infections may prevent autoimmunity (Versini et al. 2015). Previous case reports and observational studies have suggested that EBV infection may be a trigger for AIH, but no relevant basic studies or multicenter cohort studies have demonstrated this view.We demostrated that anti-EBV EBNA-1 antibodies may be a protective factor against autoimmune hepatitis.EBNA-1 deems a major nuclear protein expressed in all EBV-infected cells (Yadav et al. 2016). It plays a critical role in efficient EBV genome replication, persistence, and transcription in dividing cells by binding to the replication origin (oriP) of EBV, ensuring stable maintenance of the viral genome during cell division (Reisman et al. 1985). EBNA-1 is uniquely expressed in both latent and lytic infection states, and regulates gene expression during the latent phase by binding to oriP. Studies have shown that EBNA-1 interacts with viral DNA elements and cellular promoters, upregulating STAT1 (signal transducer and activator of transcription 1) and downregulating the tumor growth factor β (TGF-β) signaling pathway, leading to a decrease in SMAD2 and inhibition of the canonical NF-κB pathway through the suppression of IKK (IκB kinase) phosphorylation (Baumforth et al. 2008). The NF-κB pathway is a key regulator of inflammation and has been implicated in the pathogenesis of autoimmune hepatitis. Activation of the NF-κB pathway promotes the secretion of various inflammatory factors, exacerbating the inflammatory response in AIH. Conversely, inhibition of the NF-κB pathway can alleviate this inflammatory response and consequently improve AIH symptoms (Kang et al. 2023). The observed inhibition of the NF-κB pathway by EBNA-1 may explain the protective effects of EBV infection against AIH.

Simultaneously, our reverse Mendelian randomization analysis indicated that genetically determined AIH may serve as a protective factor against elevated levels of anti-EBV EBNA-1 antibodies. Notably, there is a paucity of global research on the influence of AIH on EBV infection. An observational study reported several cases of acute EBV infection in patients with AILD who were undergoing treatment with immunosuppressive agents, whereas no such cases were observed in the non-immunosuppressive group (Nayagam et al. 2022). This suggests that enhanced immune activity in the AILD state may have inhibitory effects on EBV infection.

Furthermore, our multi-layered genetic dissection revealed distinct causal architectures linking EBV antigen-specific responses to autoimmune liver pathologies, and five EBNA-1-associated genes demonstrated causal relationships with AIH. Gene enrichment analysis revealed significant involvement of MAP-kinase scaffold activity, a pathway previously implicated in AIH progression, where its inhibition has been shown to ameliorate disease severity (Liu et al. 2023; Ma et al. 2007). Immunophenotyping demonstrated marked B cell expansion in patients with AIH compared to controls, a finding corroborated by single-cell RNA sequencing that specifically identified elevated AIF1-positive B cell populations. Although the precise role of B cells in AIH pathogenesis remains unclear, our findings suggest a potential mechanistic link through AIF1 (allograft inflammatory factor 1)—a key regulator of phagocytosis, membrane ruffling, and F-actin polymerization, which has been associated with multiple autoimmune disorders (De Leon-Oliva et al. 2023). Previous studies have established that AIF1 is involved in modulating B cell function during inflammatory responses, with a demonstrated capacity to suppress immune activation through B cell inhibition (Spaargaren et al. 2003). These results implicate AIF1-positive B cells are potentially critical mediators in AIH progression, warranting further investigation into this cellular population and its therapeutic implications.

Meanwhile, our study suggests that increased levels of anti-EBV ZEBRA antibodies are a causative risk factor for PBC.Previous studies have shown that EBV-DNA levels are elevated in the peripheral blood mononuclear cells of PBC patients, and EBV early antigen titers are increased in PBC (Jiang et al. 2022). However, there is an absence of definitive evidence to establish a conclusive link between PBC and EBV. ZEBRA (also termed Z, EB-1, Zta, or BZLF1), which is encoded by the EBV BZLF1 gene, activates the entire EBV lytic cycle cascade when present in latently infected cells (Wu et al. 2024). It has been demonstrated to activate early genes involved in metabolism and viral DNA replication as well as late proteins that are responsible for the structure of EBV. EBV reactivation has been found to be strongly correlated with ZEBRA (Yuming et al. 2024).

Our analysis identified four ZEBRA-responsive genes that exhibit specific associations with PBC, including TGFBR2, a well-established mediator in spontaneous PBC mouse models. Enrichment analysis revealed that these genes are closely linked to estrogen-responsive pathways and TGF-β superfamily signaling, and previous studies have demonstrated that EBV reactivation elevates numerous inflammatory mediators, including IL-6, IL-8, IL-10, IL-13, and TGF-β (Jones et al. 2007; Morales-Sanchez & Fuentes-Panana 2018). Notably, ZEBRA enhances the expression of TGF-β and VEGF genes in B cells during the lytic cycle (Katsumura et al. 2012). Furthermore, ZEBRA stimulates the early lytic gene BLLF3 to activate NF-κB, triggering the secretion of pro-inflammatory cytokines (TNF-α, IL-1β, IL-6, IL-8, and IL-10) from human monocyte-derived macrophages (Zheng et al. 2023). Consistent with other autoimmune disorders, PBC pathogenesis involves elevated inflammatory factors (IL-6, IL-8, IL-10, TGF-β) and immune cell infiltration, particularly CD4 + and CD8 + T lymphocytes (Lieshout et al. 2023). Our immune profiling confirmed significant enrichment of M1 macrophages in PBC patients, suggesting enhanced inflammatory responses during disease progression.These findings support a model whereby EBV infection of biliary epithelial cells, through ZEBRA reactivation, drives inflammatory cytokine release that may accelerate PBC development.

In summary, our study demonstrated a strong association between EBV infection and AILDs. Nevertheless, this study is not without its limitations, which require careful consideration. First, the data primarily originated from individuals of European ancestry and were constrained to adults, with no stratification by sex or age. These factors may have impacted the external validity and precision of the findings. Second, although our screening process identified relevant diagnostic genes between EBV and AILDs, the differential expression and LASSO regression analysis for AIH were performed on a very small dataset (GSE159676: 3 AIH vs. 6 controls), which limits the statistical power and increases the risk of model overfitting, as indicated by the high AUC value. These findings should therefore be considered exploratory and hypothesis-generating, requiring validation in larger, independent cohorts.Third, it is important to note that our scRNA-seq analysis was performed on PBMCs rather than liver tissue. While this provides insights into the systemic immune response, future studies on liver-specific immune infiltrates are warranted.Future studies utilizing liver-specific eQTL data from GTEx or other sources are essential to confirm and refine the gene-level associations identified here.Finally, it should be emphasized that the instrumental variables of this study reflect the genetic predisposition to antibody reactivity rather than antibody levels per se, and that actual antibody levels are influenced by multiple non-genetic factors. Future studies need to be validated on datasets that more directly measure EBV exposure and antibody status and to explore in depth its underlying immunological mechanisms.

## Conclusion

In conclusion, our study established a novel genetic interplay between Epstein-Barr virus infection and autoimmune liver diseases, identifying pivotal molecular signatures and disease-associated immune cell populations. These findings highlight the need for further mechanistic studies to elucidate the specific and clear link between EBV infection and AILDs. Understanding these mechanisms is essential to optimize surveillance and early diagnosis, ultimately leading to more effective management and treatment strategies.

## Supplementary Information

Below is the link to the electronic supplementary material.

Supplementary Material 1

Supplementary Material 2

## Data Availability

The Data of antibodies against EBV for the present study can be downloaded from GWAS (GWAS ID included in the article), and the GWAS data of AILDs were obtained from the FinnGen database (https://www.finngen.fi/en). GEO data (GSE159676, GSE61260, and GSE186333) were obtained from the GEO website (https://www.ncbi.nlm.nih.gov/geo). Further inquiries can be directed to the corresponding authors.The code supporting the findings of this study is publicly available on GitHub (https://github.com/csuzly226/Multi-omics-Profiling-Uncovers-Paradoxical-EBV-Involvement-in-Autoimmune-Liver-Disease-Pathogenesis.git).
