# Supplementary material for: Multi-omics profiling uncovers paradoxical Epstein-Barr virus involvement in autoimmune liver disease pathogenesis
Source: AMB Express. 2025 Oct 27;15:156. doi: 10.1186/s13568-025-01975-6 (PMC12559547; doi:10.1186/s13568-025-01975-6)
Supplement: Supplementary file 1 — Supplementary Material 1 [file 13568_2025_1975_MOESM1_ESM.docx]

Supplementary Materials

**The PDF file includes:**

Figs. S1 to S29

**Other Supplementary Material for this manuscript includes the following:**

Tables S1 and S20


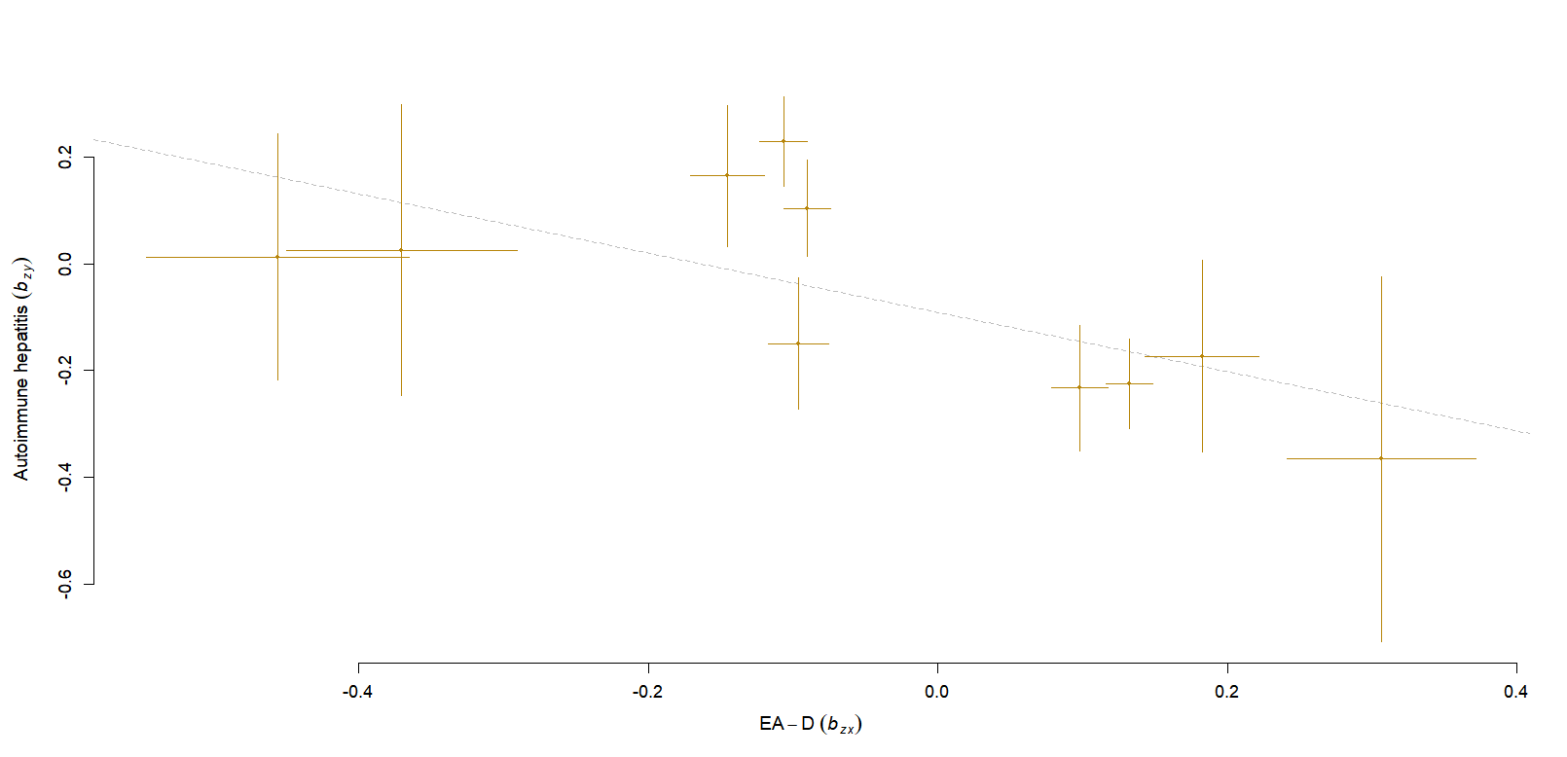


**Fig. S1. GSMR analysis of anti-EBV EA-D antibodies with autoimmune hepatitis.** Scatter plots of GSMR analysis report the causal association with antibodies of EBV over autoimmune liver disease. HEIDI-outlier approach was utilized to remove instrumental outliers and eliminate pleiotropic SNPs. Linear relation of standardized exposure estimates (*bzx*) on outcome estimates (*bzy*) was displayed in the plots.


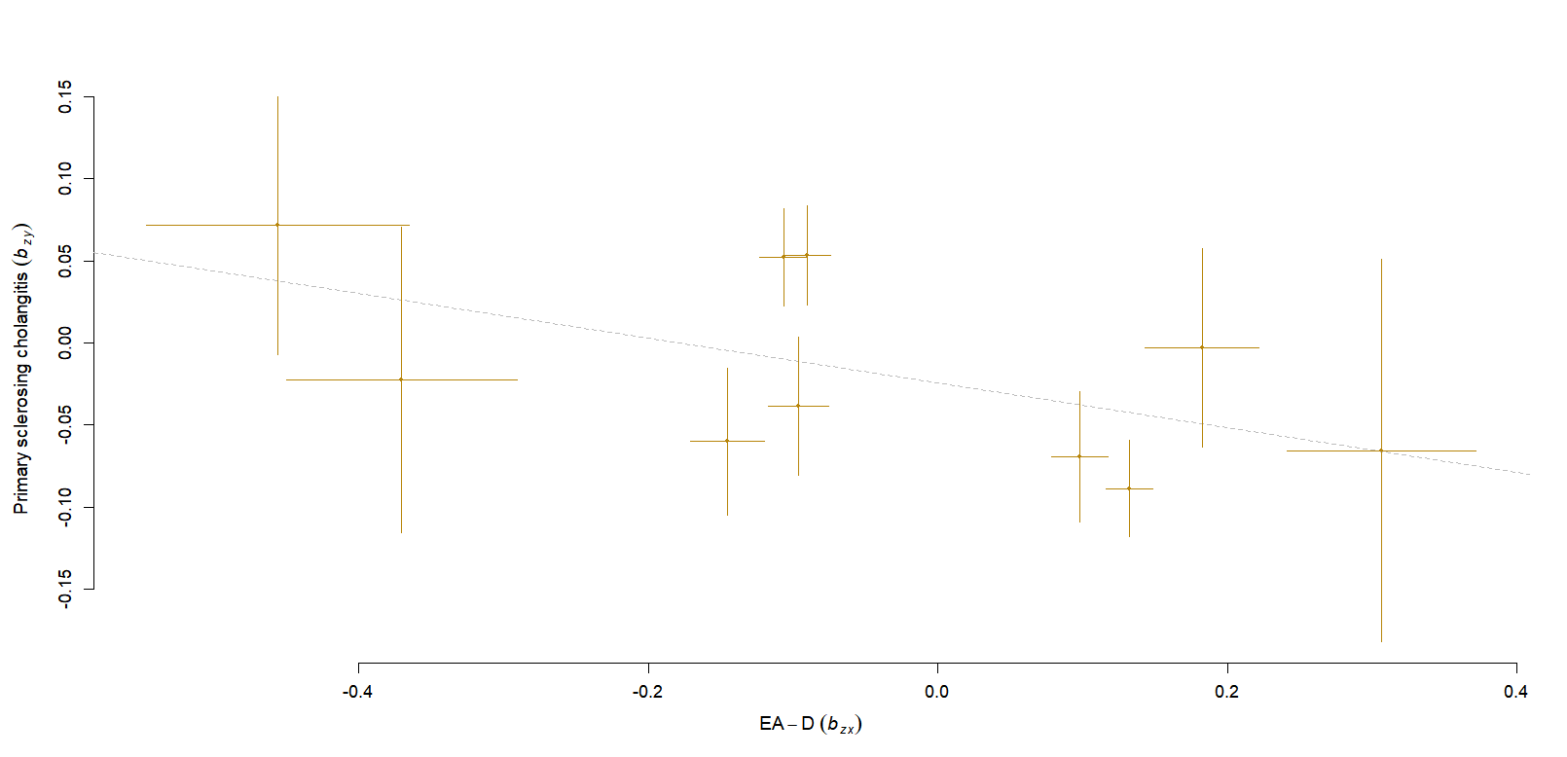


**Fig. S2. GSMR analysis of anti-EBV EA-D antibodies with primary sclerosing cholangitis.** Scatter plots of GSMR analysis report the causal association with antibodies of EBV over autoimmune liver disease. HEIDI-outlier approach was utilized to remove instrumental outliers and eliminate pleiotropic SNPs. Linear relation of standardized exposure estimates (*bzx*) on outcome estimates (*bzy*) was displayed in the plots.


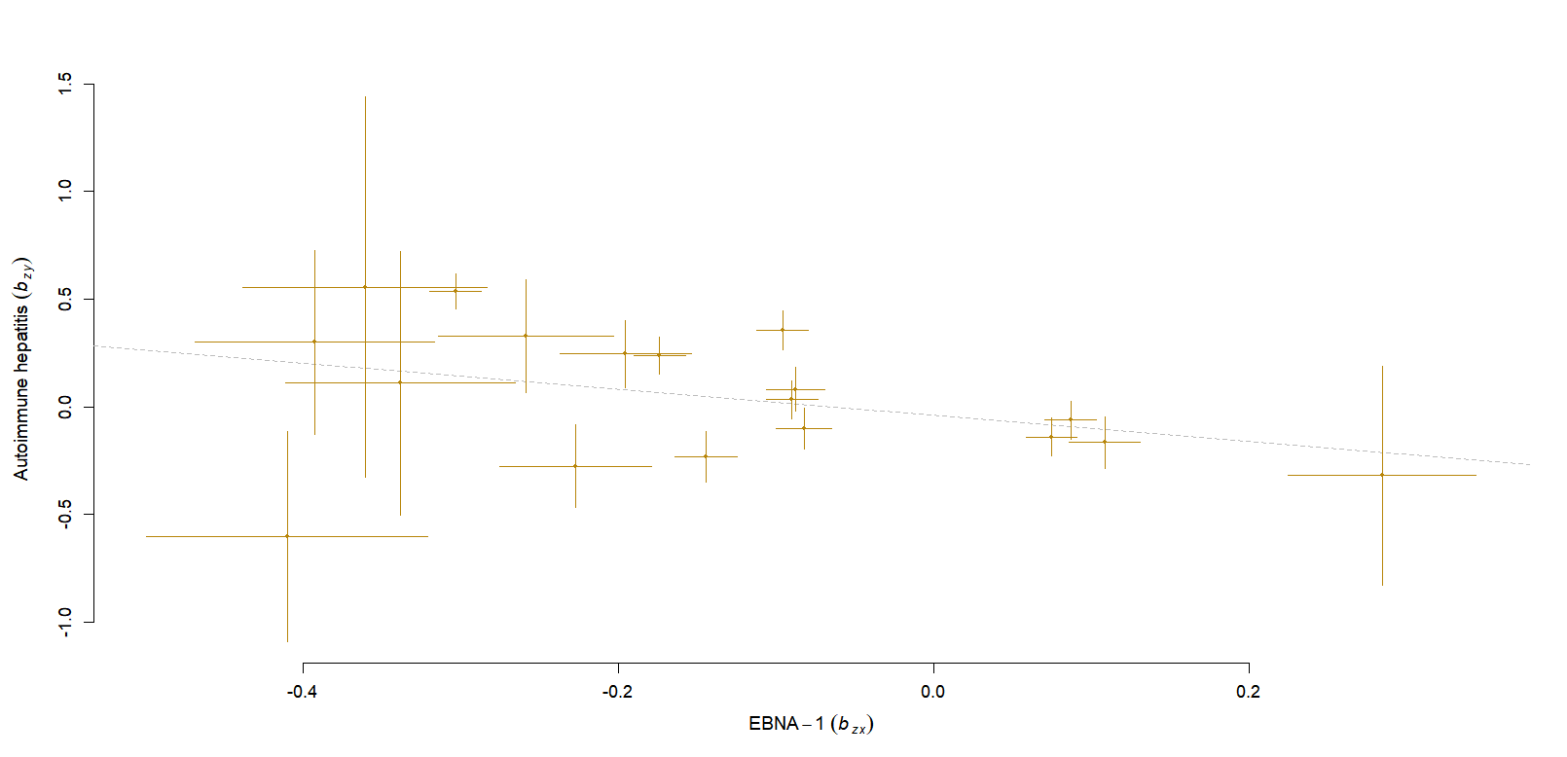


**Fig. S3. GSMR analysis of anti-EBV EBNA-1 antibodies with autoimmune hepatitis.** Scatter plots of GSMR analysis report the causal association with antibodies of EBV over autoimmune liver disease. HEIDI-outlier approach was utilized to remove instrumental outliers and eliminate pleiotropic SNPs. Linear relation of standardized exposure estimates (*bzx*) on outcome estimates (*bzy*) was displayed in the plots.


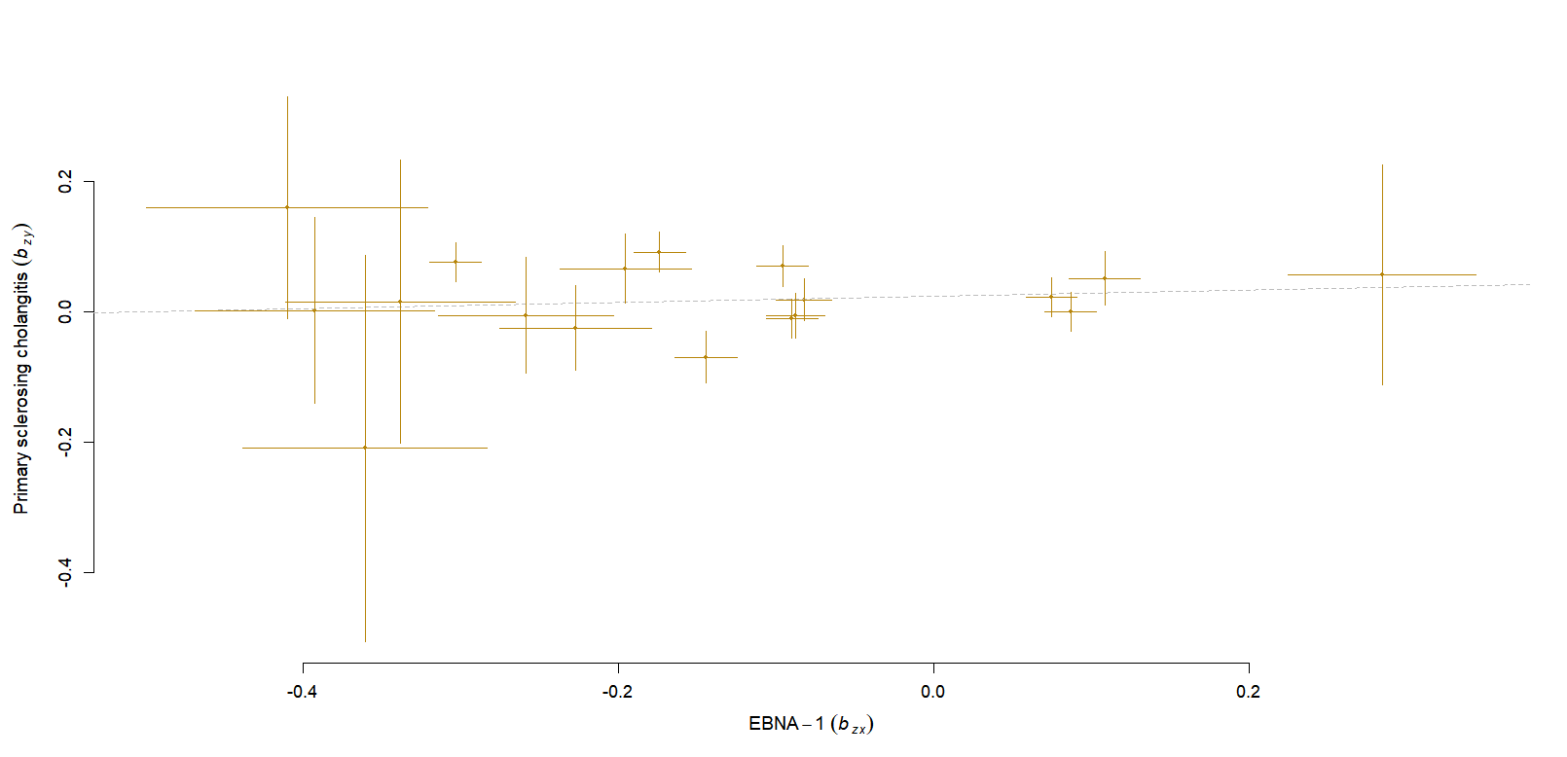


**Fig. S4. GSMR analysis of anti-EBV EBNA-1 antibodies with primary sclerosing cholangitis.** Scatter plots of GSMR analysis report the causal association with antibodies of EBV over autoimmune liver disease. HEIDI-outlier approach was utilized to remove instrumental outliers and eliminate pleiotropic SNPs. Linear relation of standardized exposure estimates (*bzx*) on outcome estimates (*bzy*) was displayed in the plots.


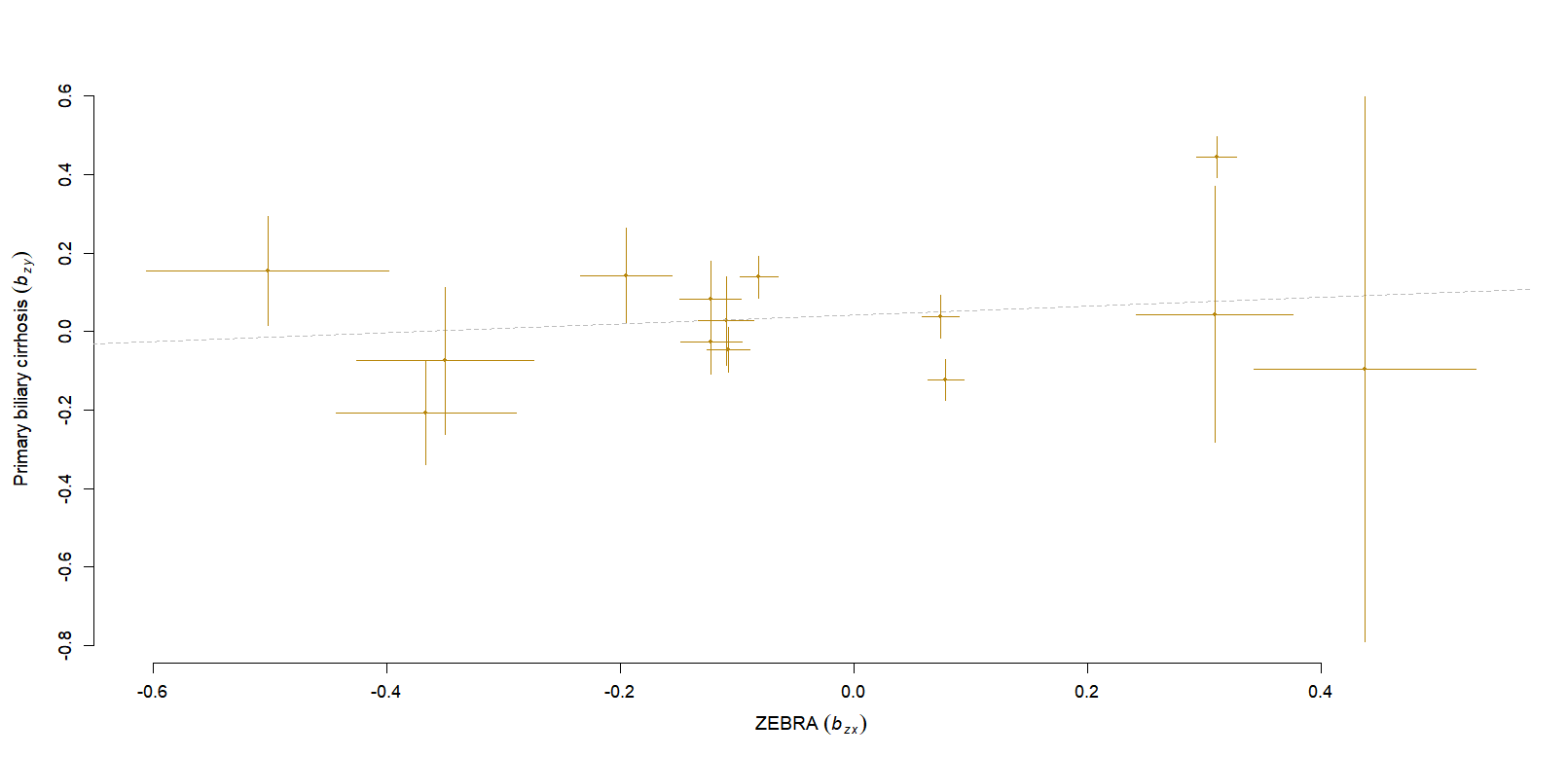


**Fig. S5. GSMR analysis of anti-EBV ZEBRA antibodies with primary biliary cholangitis.** Scatter plots of GSMR analysis report the causal association with antibodies of EBV over autoimmune liver disease. HEIDI-outlier approach was utilized to remove instrumental outliers and eliminate pleiotropic SNPs. Linear relation of standardized exposure estimates (*bzx*) on outcome estimates (*bzy*) was displayed in the plots.


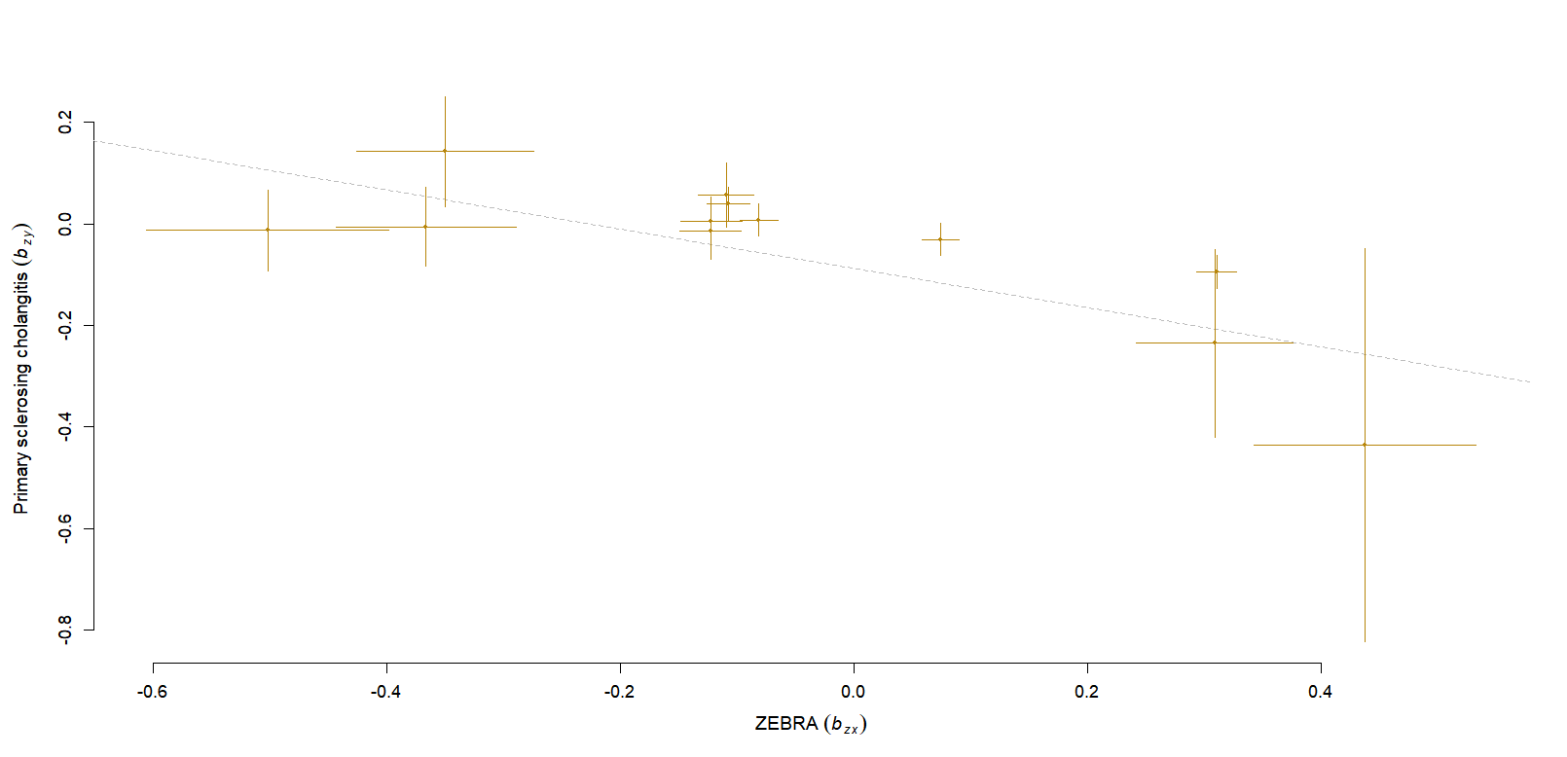


**Fig. S6. GSMR analysis of anti-EBV ZEBRA antibodies with primary sclerosing cholangitis.** Scatter plots of GSMR analysis report the causal association with antibodies of EBV over autoimmune liver disease. HEIDI-outlier approach was utilized to remove instrumental outliers and eliminate pleiotropic SNPs. Linear relation of standardized exposure estimates (*bzx*) on outcome estimates (*bzy*) was displayed in the plots.


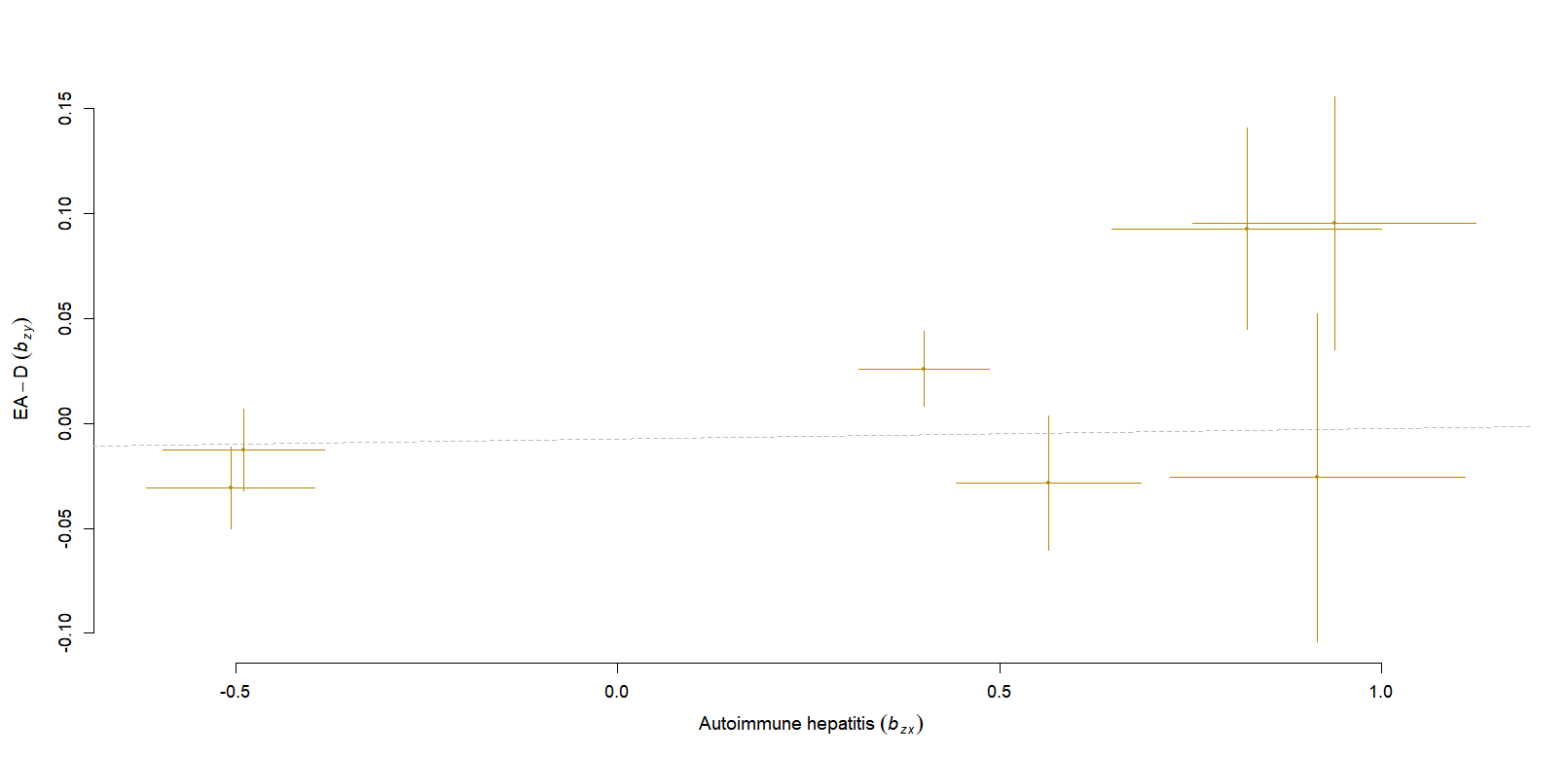


**Fig. S7. GSMR analysis of autoimmune hepatitis with anti-EBV EA-D antibodies.** Scatter plots of GSMR analysis report the causal association with autoimmune liver disease over antibodies of EBV. HEIDI-outlier approach was utilized to remove instrumental outliers and eliminate pleiotropic SNPs. Linear relation of standardized exposure estimates (*bzx*) on outcome estimates (*bzy*) was displayed in the plots.


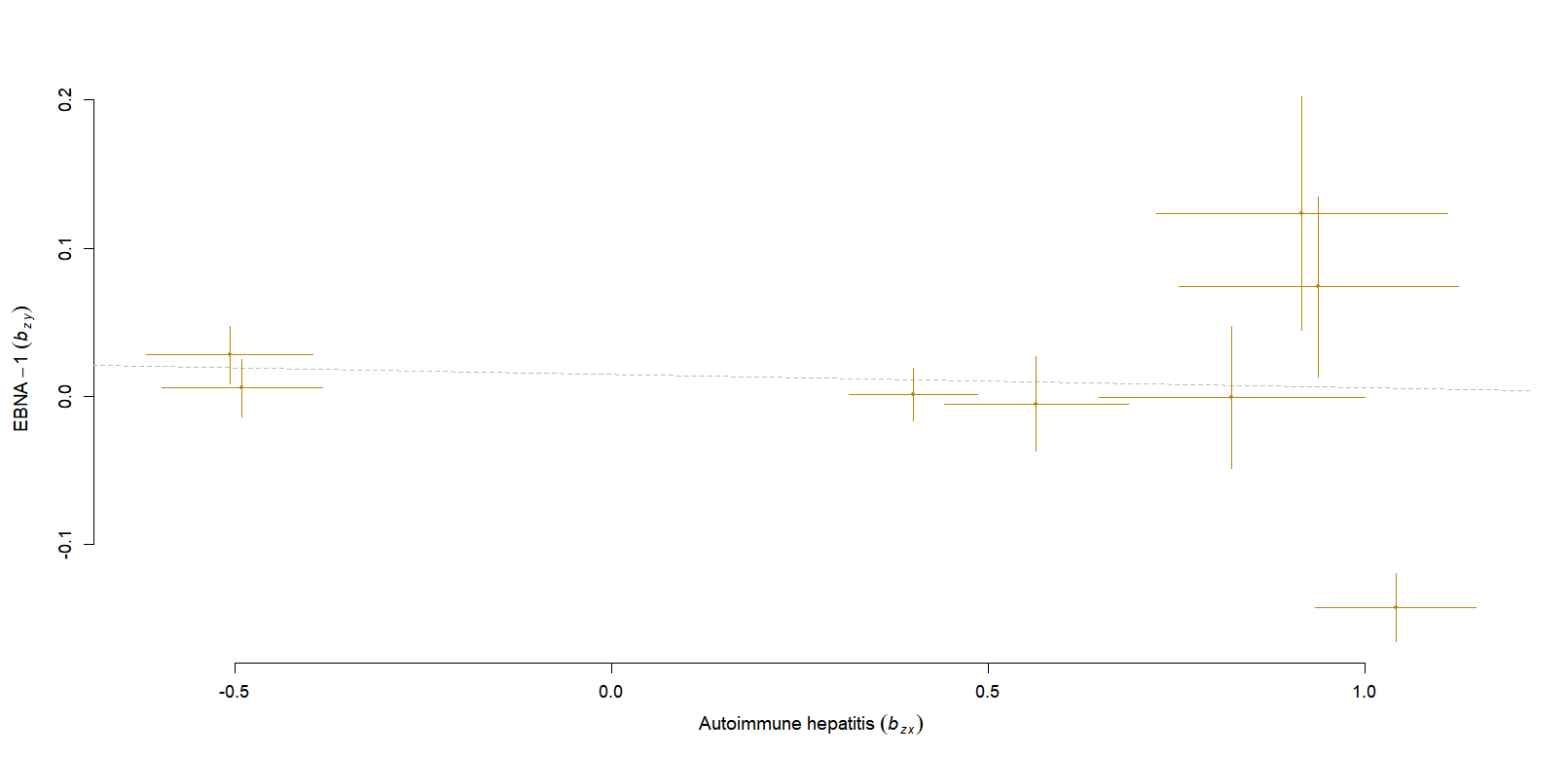


**Fig. S8. GSMR analysis of autoimmune hepatitis with anti-EBV EBNA-1 antibodies.** Scatter plots of GSMR analysis report the causal association with autoimmune liver disease over antibodies of EBV. HEIDI-outlier approach was utilized to remove instrumental outliers and eliminate pleiotropic SNPs. Linear relation of standardized exposure estimates (*bzx*) on outcome estimates (*bzy*) was displayed in the plots.


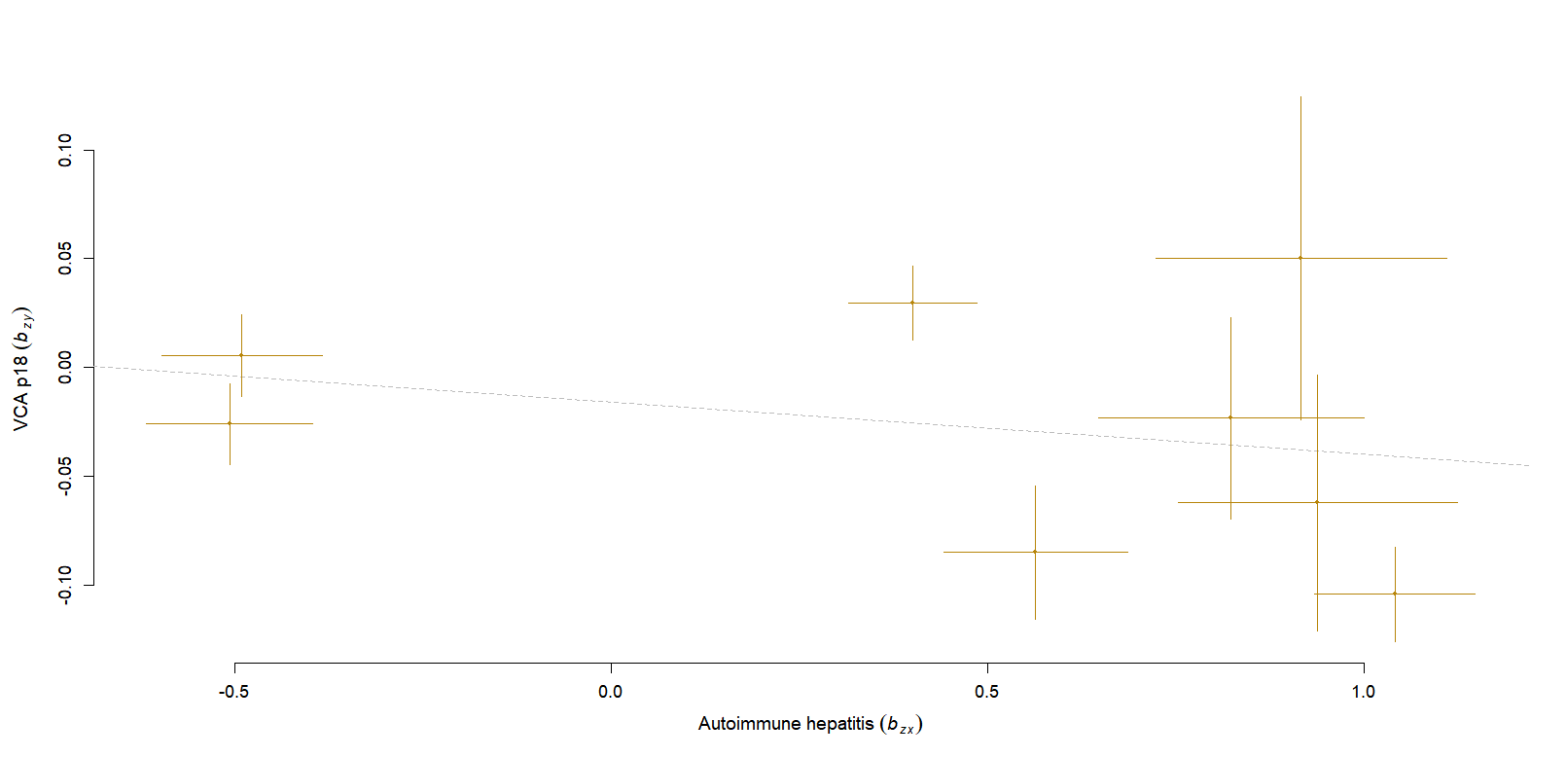


**Fig. S9. GSMR analysis of autoimmune hepatitis with anti-EBV VCA p18 antibodies.** Scatter plots of GSMR analysis report the causal association with autoimmune liver disease over antibodies of EBV. HEIDI-outlier approach was utilized to remove instrumental outliers and eliminate pleiotropic SNPs. Linear relation of standardized exposure estimates (*bzx*) on outcome estimates (*bzy*) was displayed in the plots.


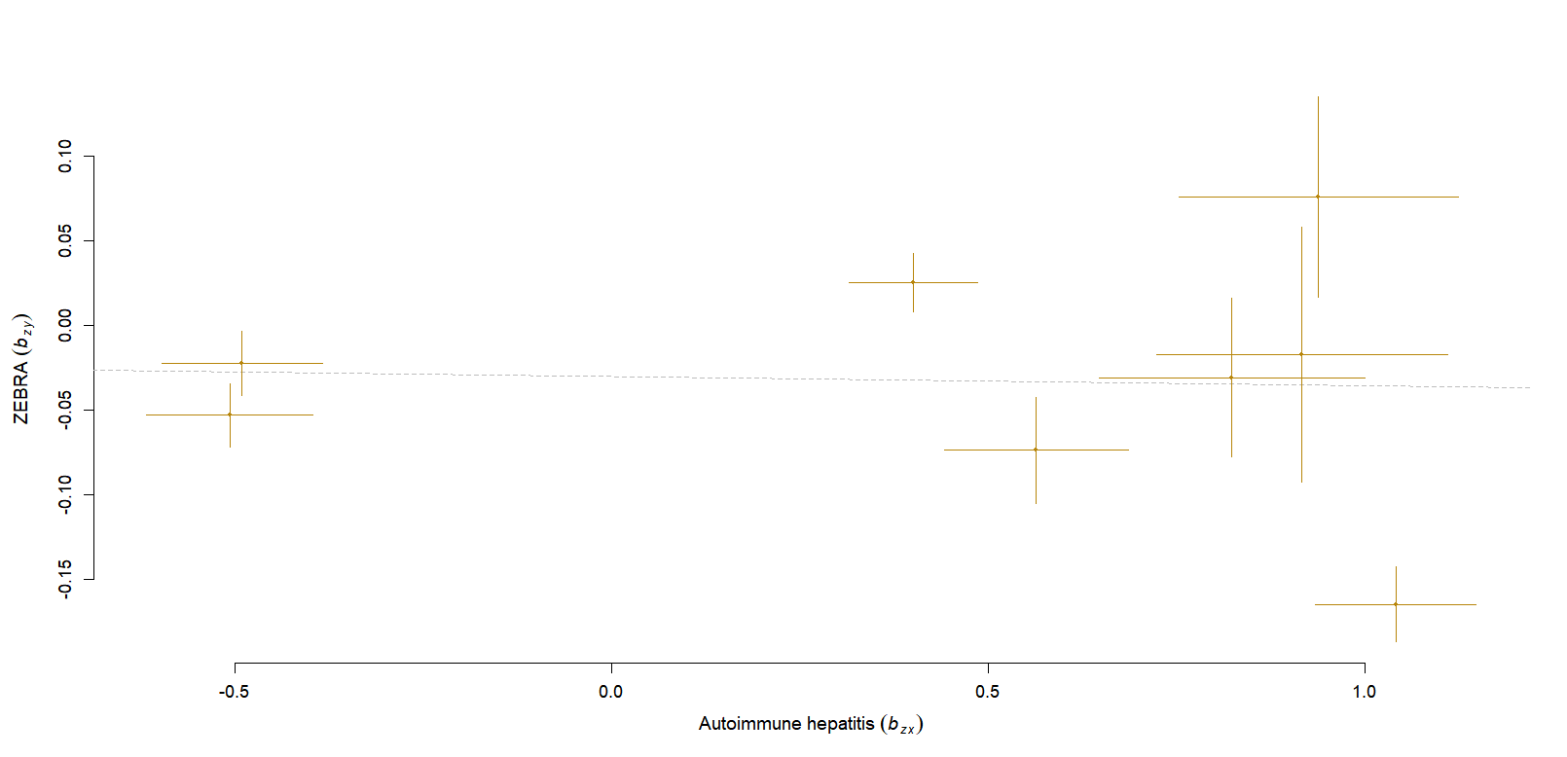


**Fig. S10. GSMR analysis of autoimmune hepatitis with anti-EBV ZEBRA antibodies.** Scatter plots of GSMR analysis report the causal association with autoimmune liver disease over antibodies of EBV. HEIDI-outlier approach was utilized to remove instrumental outliers and eliminate pleiotropic SNPs. Linear relation of standardized exposure estimates (*bzx*) on outcome estimates (*bzy*) was displayed in the plots.


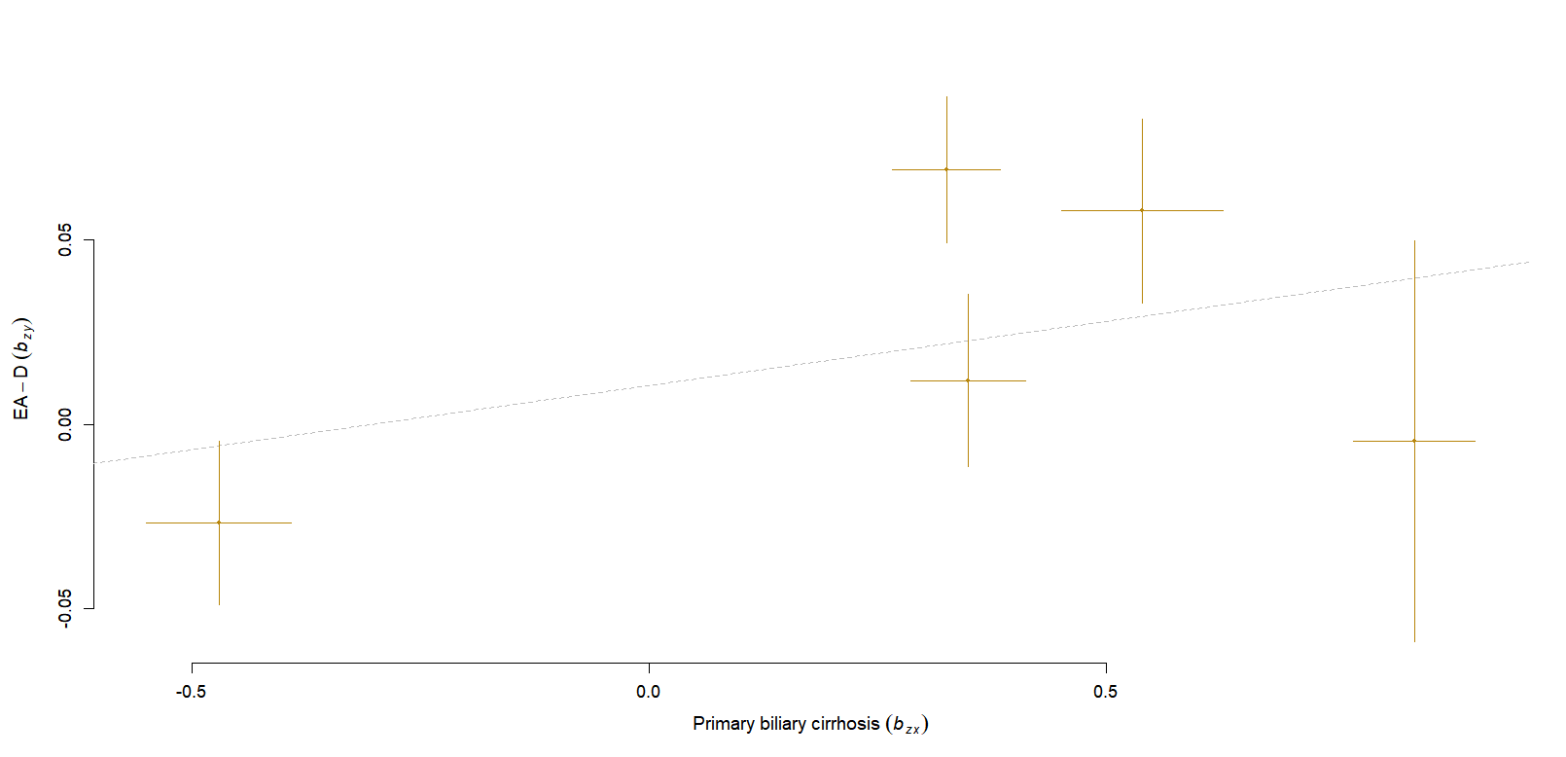


**Fig. S11. GSMR analysis of primary biliary cholangitis with anti-EBV EA-D antibodies.** Scatter plots of GSMR analysis report the causal association with autoimmune liver disease over antibodies of EBV. HEIDI-outlier approach was utilized to remove instrumental outliers and eliminate pleiotropic SNPs. Linear relation of standardized exposure estimates (*bzx*) on outcome estimates (*bzy*) was displayed in the plots.


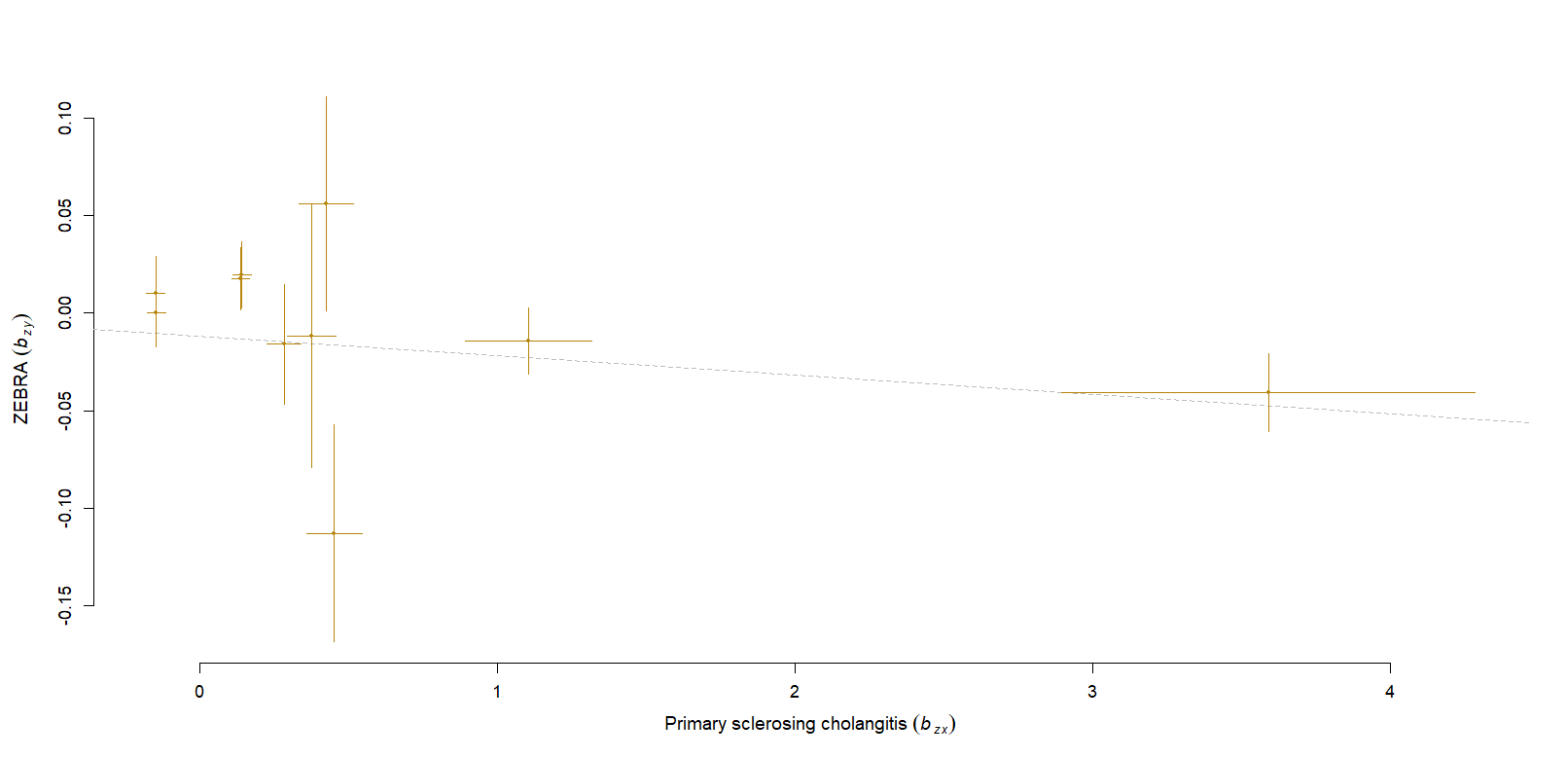


**Fig. S12. GSMR analysis of primary sclerosing cholangitis with anti-EBV ZEBRA antibodies.** Scatter plots of GSMR analysis report the causal association with autoimmune liver disease over antibodies of EBV. HEIDI-outlier approach was utilized to remove instrumental outliers and eliminate pleiotropic SNPs. Linear relation of standardized exposure estimates (*bzx*) on outcome estimates (*bzy*) was displayed in the plots.


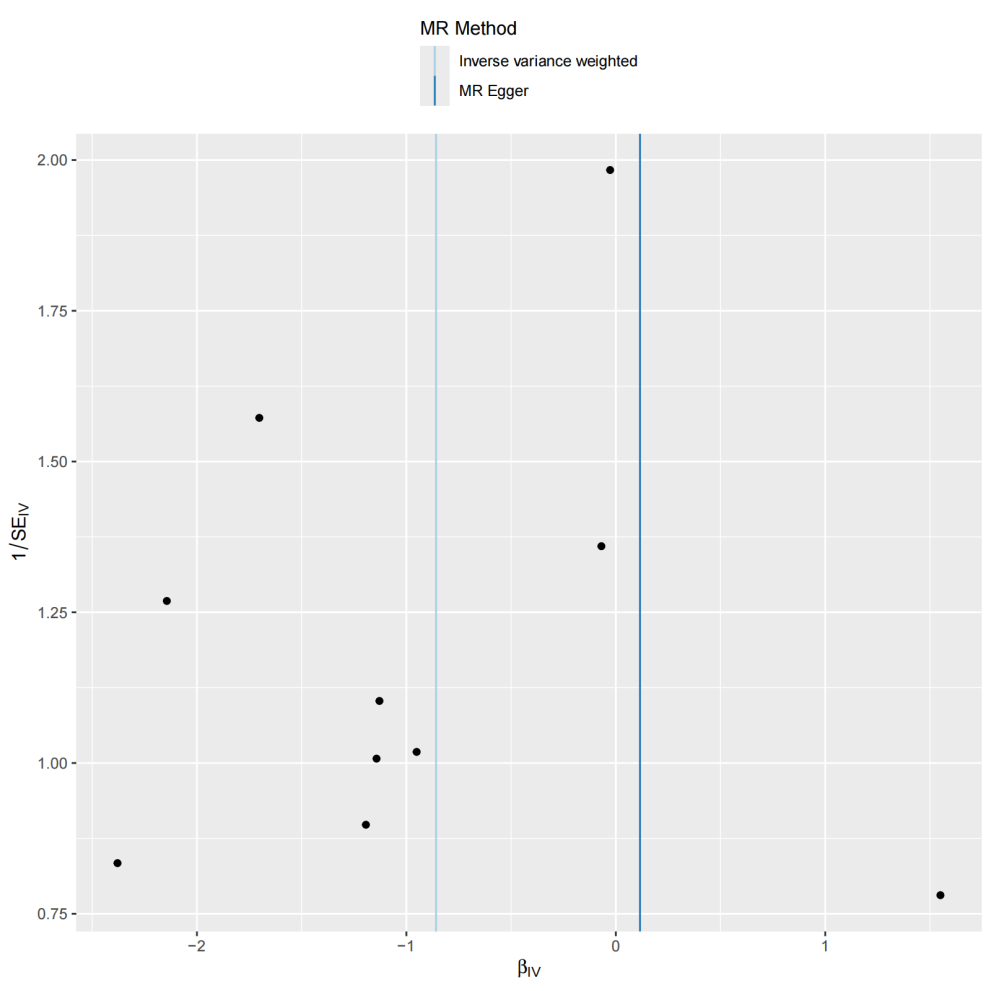


**Fig. S13. Funnel plot for the effect of anti-EBV EA-D antibodies on autoimmune hepatitis.** Funnel plot showing each SNP causal estimate against its precision.


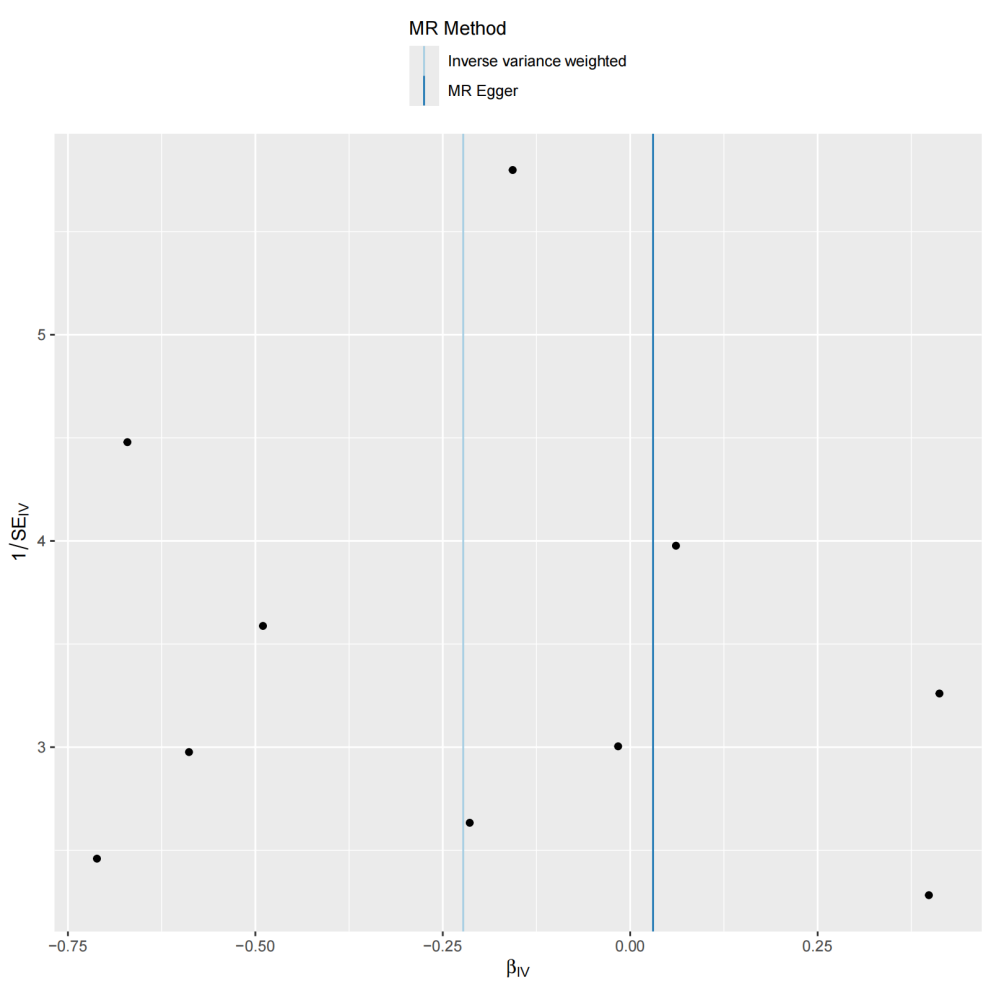


**Fig. S14. Funnel plot for the effect of anti-EBV EA-D antibodies on primary sclerosing cholangitis.** Funnel plot showing each SNP causal estimate against its precision.


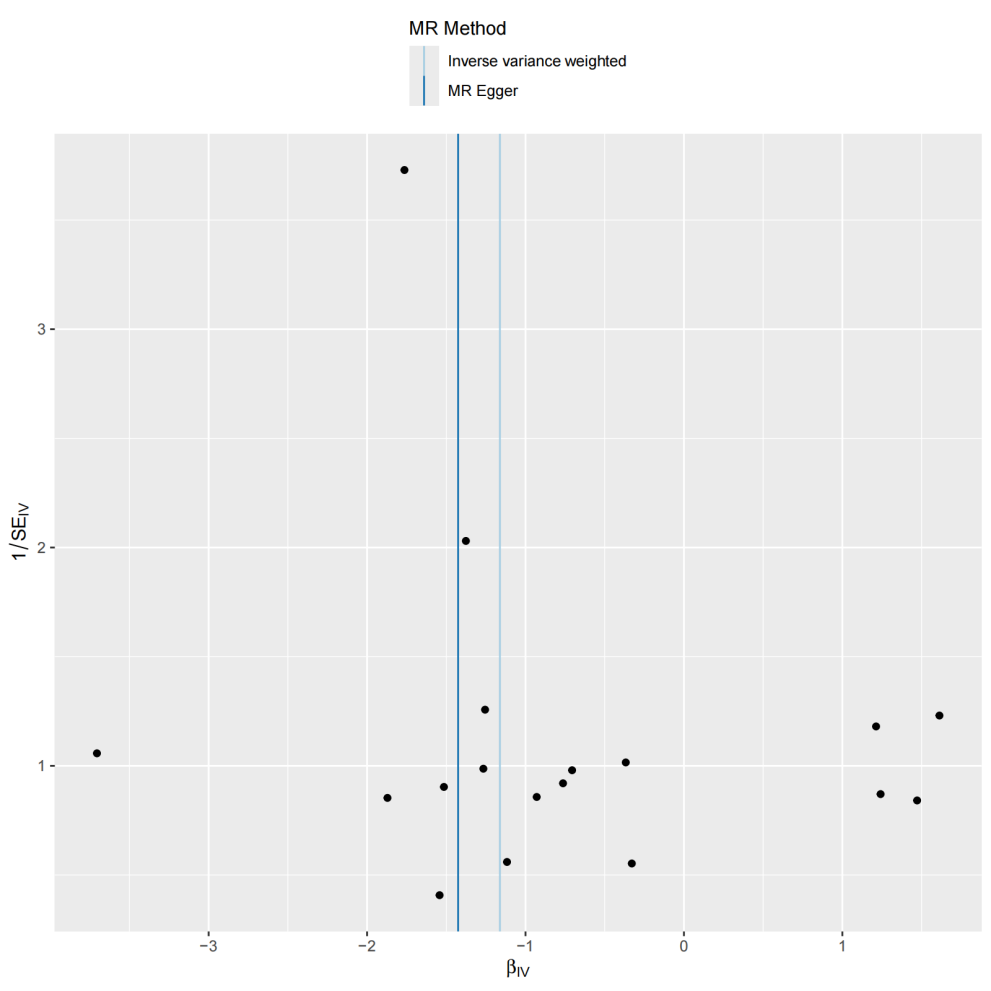


**Fig. S15. Funnel plot for the effect of anti-EBV EBNA-1 antibodies on autoimmune hepatitis.** Funnel plot showing each SNP causal estimate against its precision.


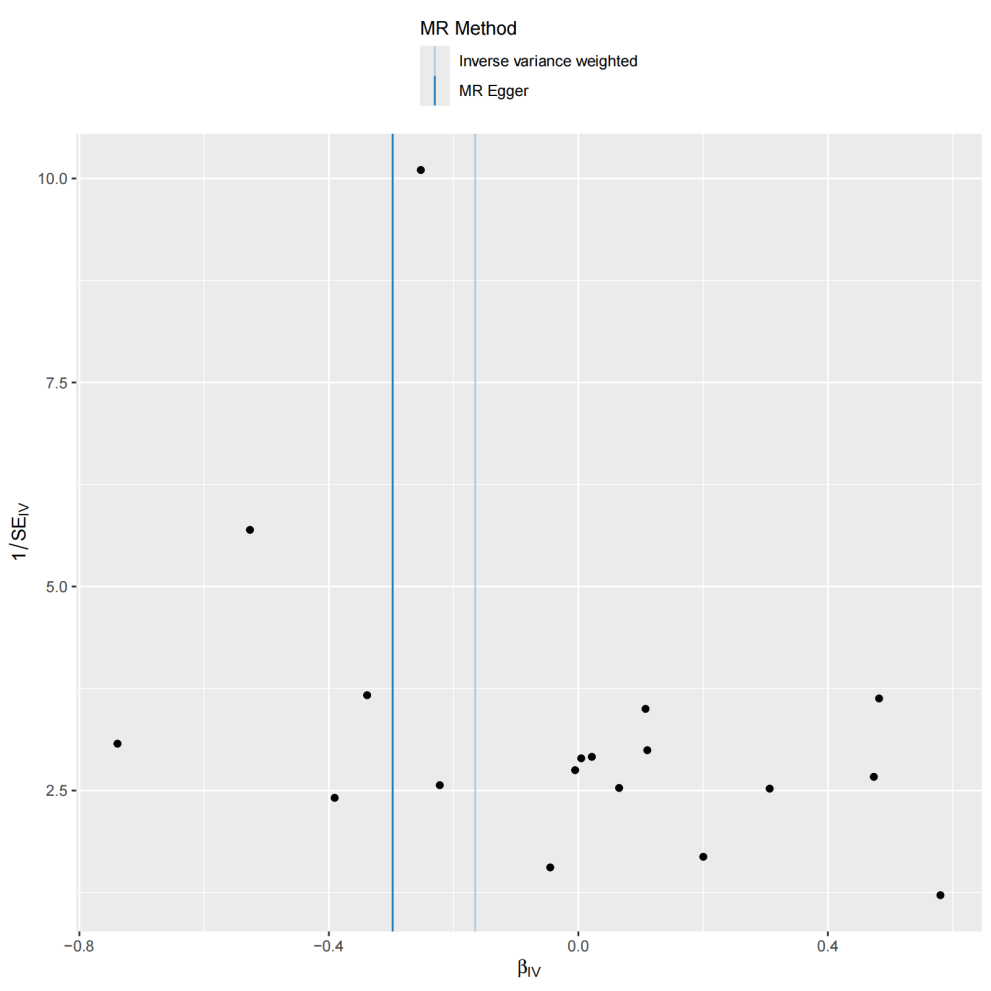


**Fig. S16. Funnel plot for the effect of anti-EBV EBNA-1 antibodies on primary sclerosing cholangitis.** Funnel plot showing each SNP causal estimate against its precision.


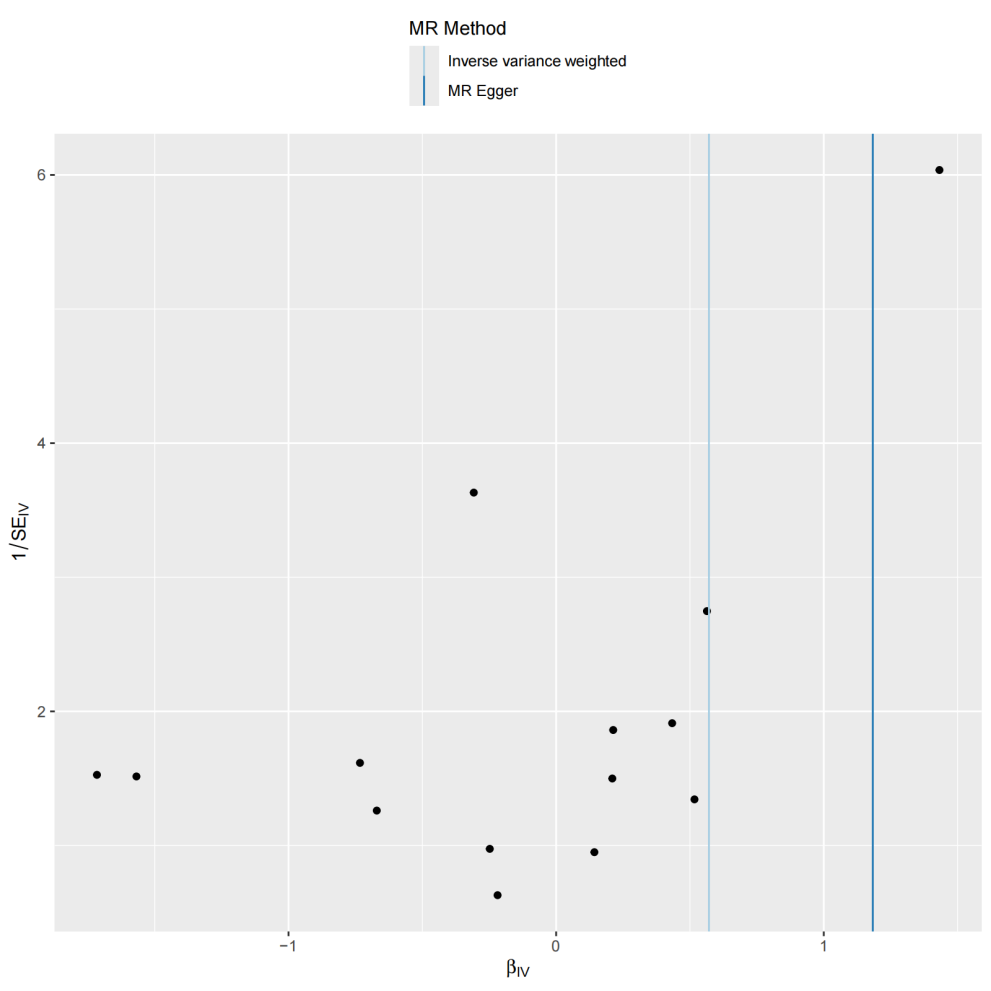


**Fig. S17. Funnel plot for the effect of anti-EBV ZEBRA antibodies on primary biliary cholangitis.** Funnel plot showing each SNP causal estimate against its precision.


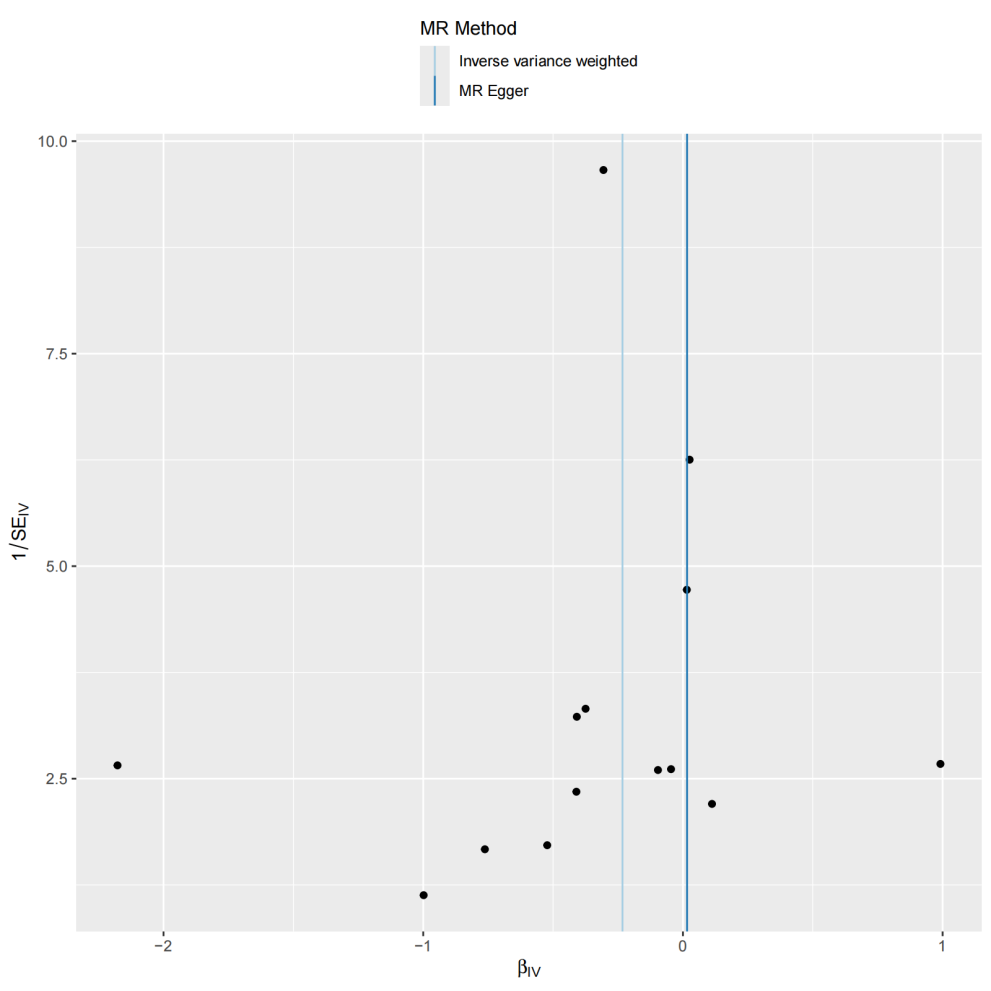


**Fig. S18. Funnel plot for the effect of anti-EBV ZEBRA antibodies on primary sclerosing cholangitis.** Funnel plot showing each SNP causal estimate against its precision.


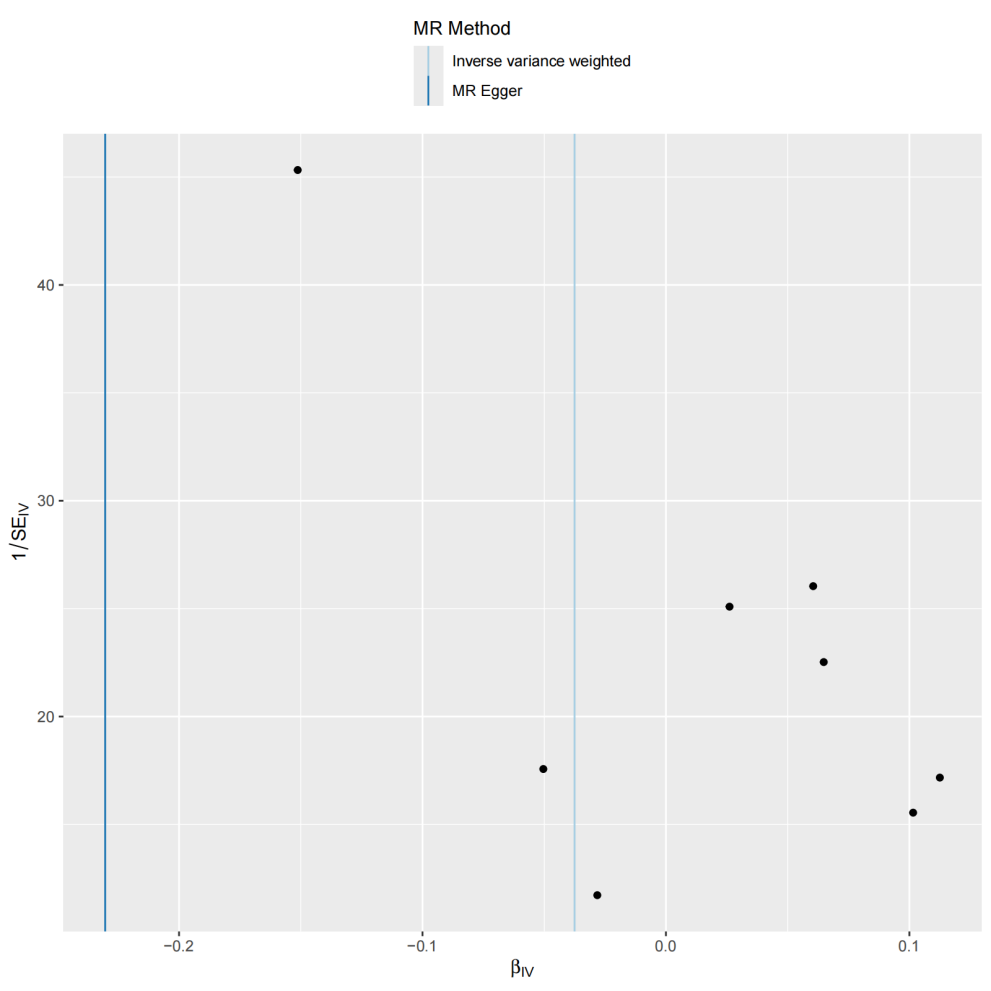


**Fig. S19. Funnel plot for the effect of autoimmune hepatitis on anti-EBV EA-D antibodies.** Funnel plot showing each SNP causal estimate against its precision.


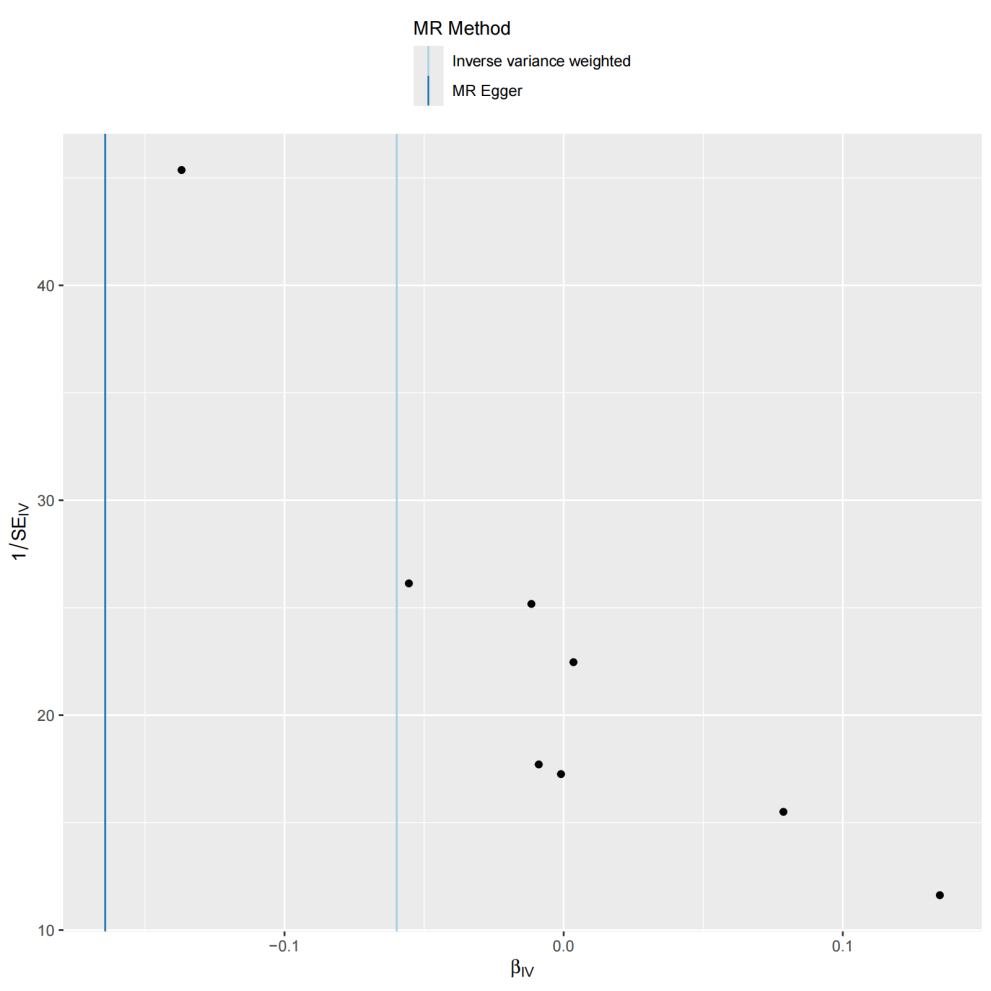


**Fig. S20. Funnel plot for the effect of autoimmune hepatitis with anti-EBV EBNA-1 antibodies.** Funnel plot showing each SNP causal estimate against its precision.


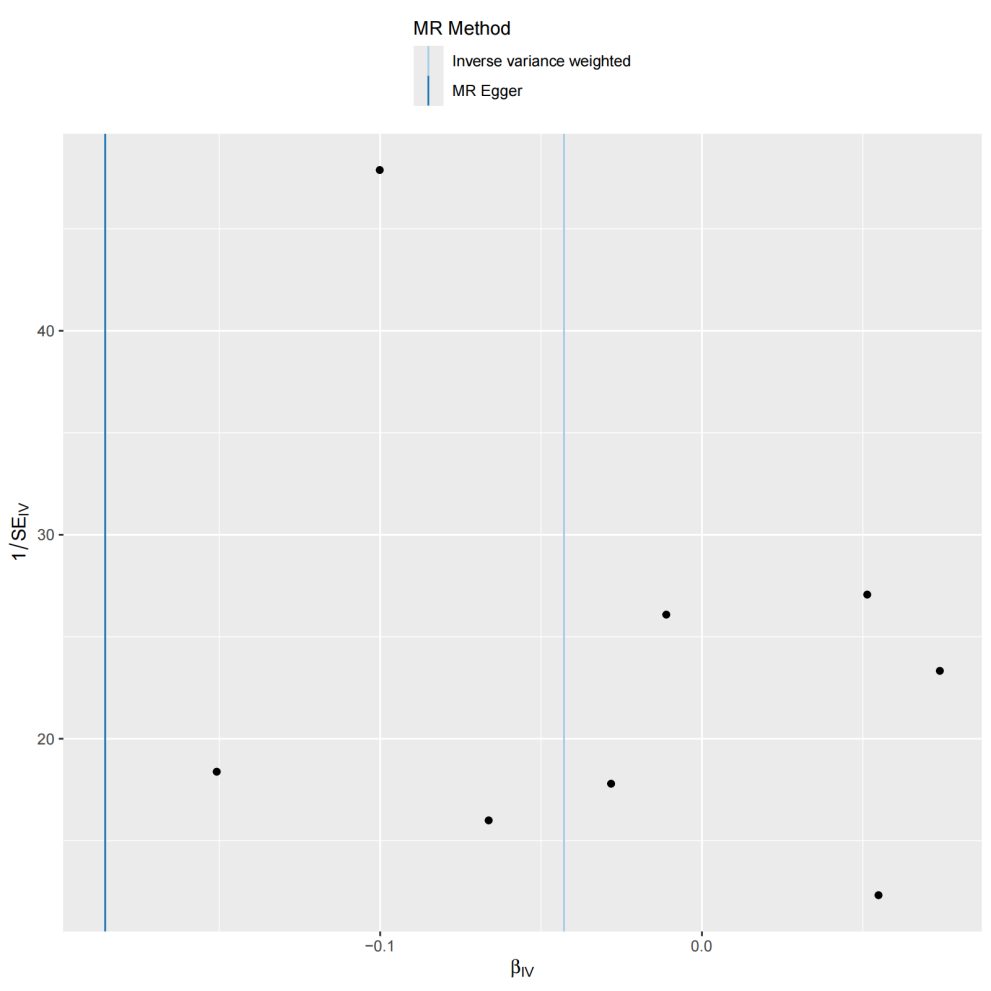


**Fig. S21. Funnel plot for the effect of autoimmune hepatitis on anti-EBV VCA p18 antibodies.** Funnel plot showing each SNP causal estimate against its precision.


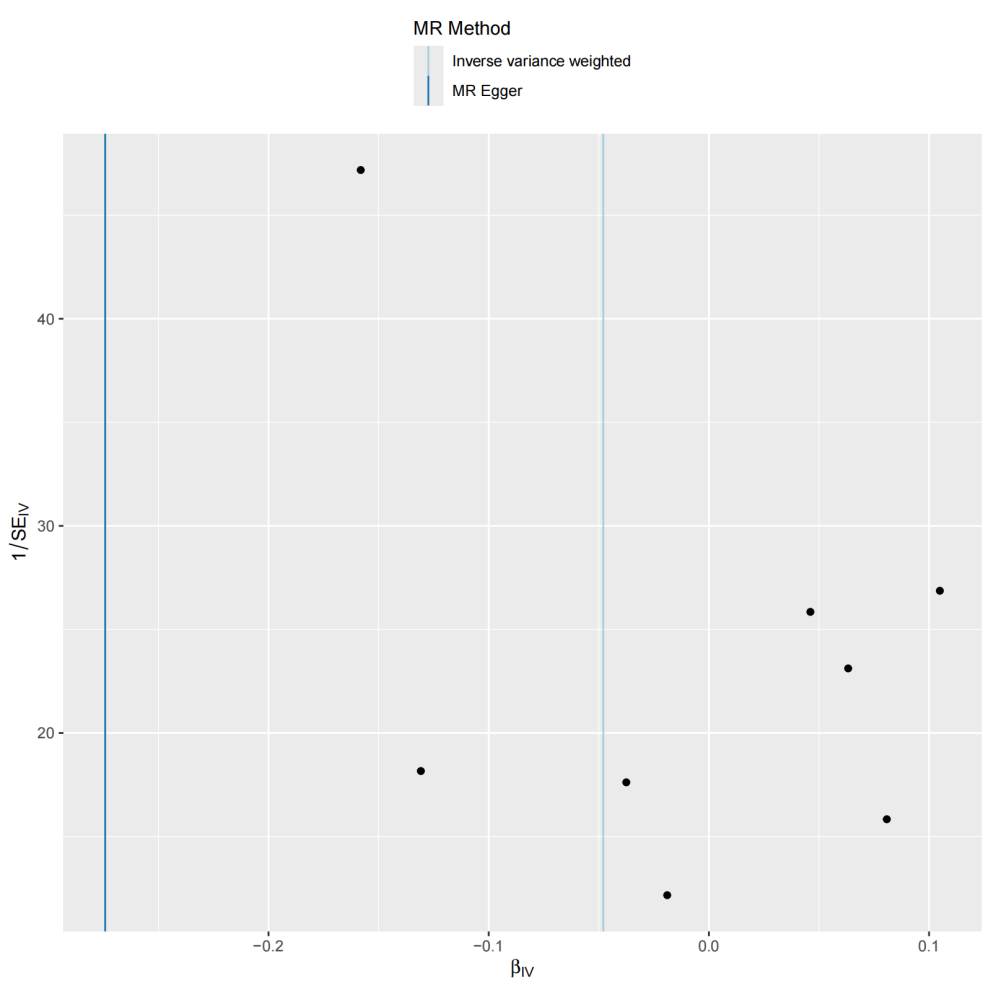


**Fig. S22. Funnel plot for the effect of autoimmune hepatitis on anti-EBV ZEBRA antibodies.** Funnel plot showing each SNP causal estimate against its precision.


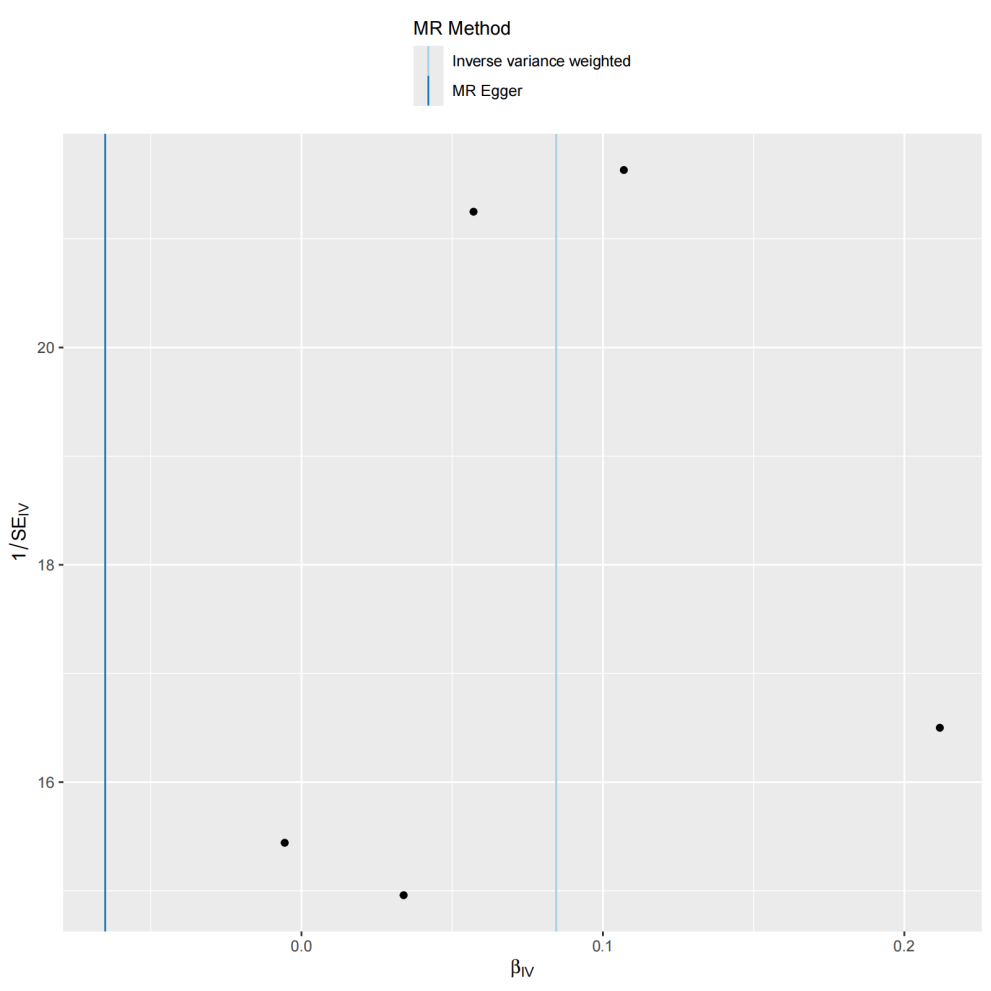


**Fig. S23. Funnel plot for the effect of primary biliary cholangitis on anti-EBV EA-D antibodies.** Funnel plot showing each SNP causal estimate against its precision.


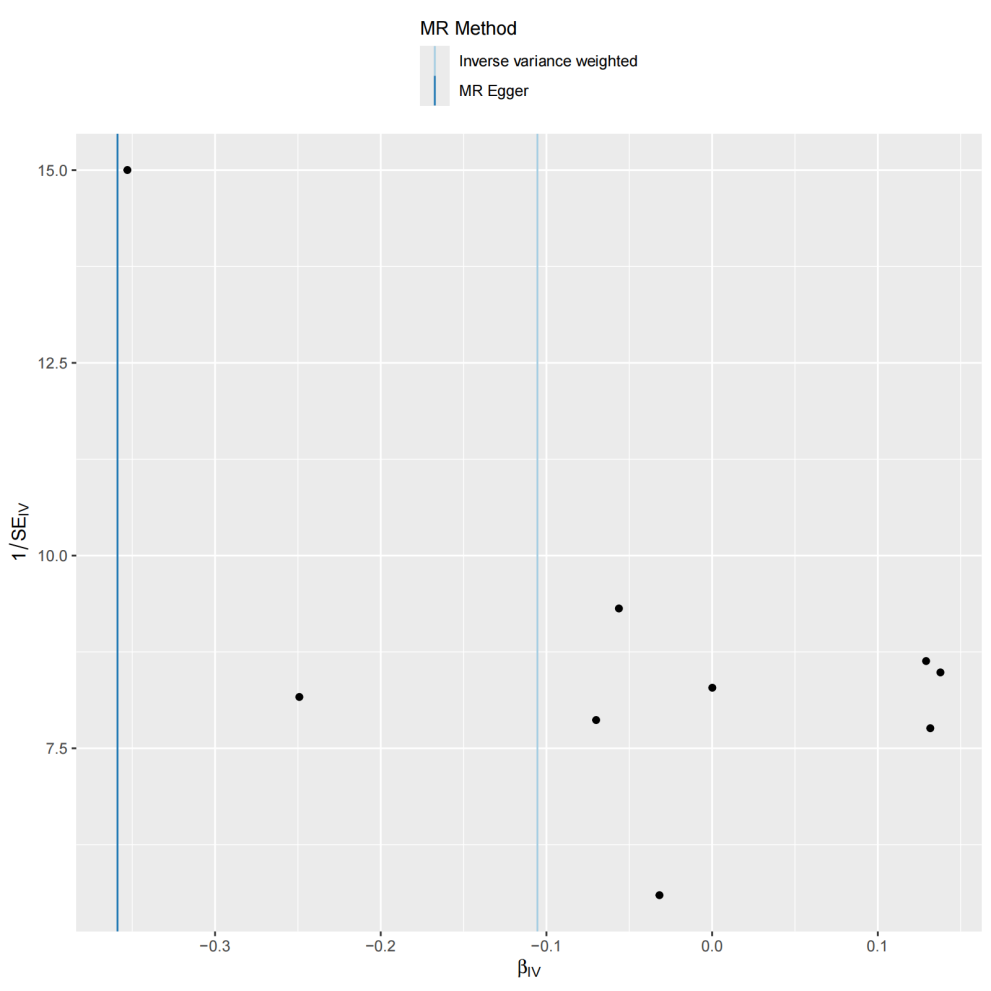


**Fig. S24. Funnel plot for the effect of primary sclerosing cholangitis on anti-EBV ZEBRA antibodies.** Funnel plot showing each SNP causal estimate against its precision.


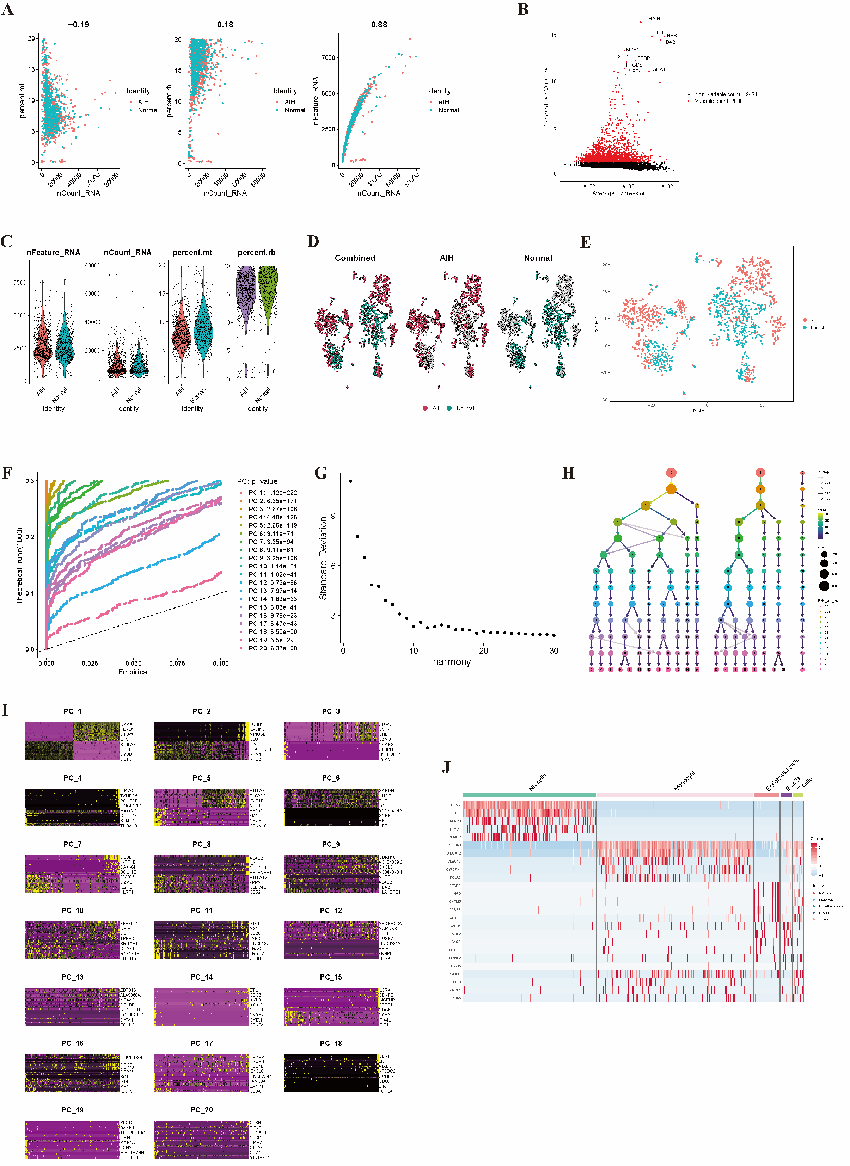


**Fig. S25. Preliminary filtering and processing of single-cell sequencing data.**


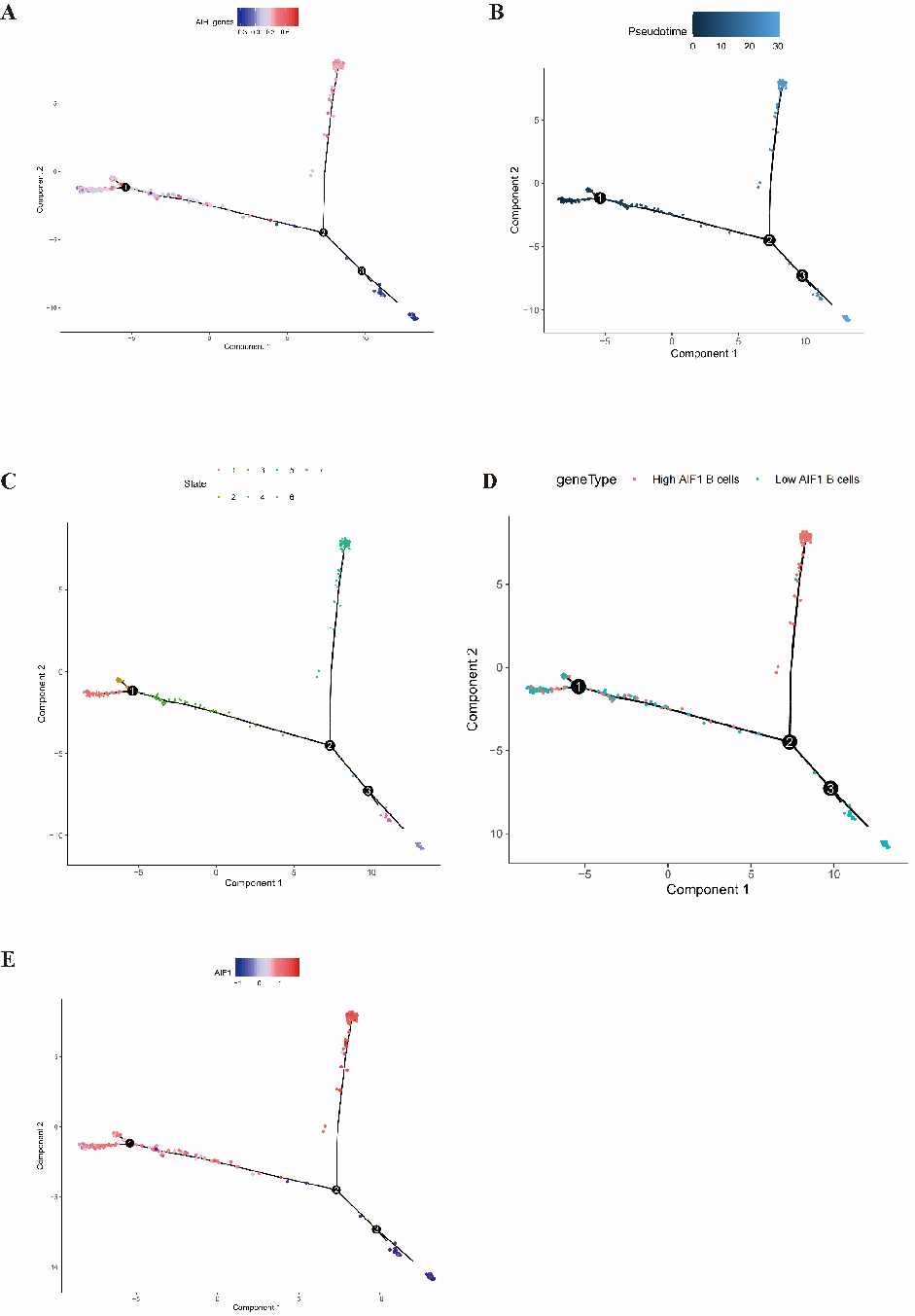


**Fig. S26. Pseudotime trajectories revealed temporal changes in AIF1-positive B cells versus AIF1-negative B cells during disease development.**


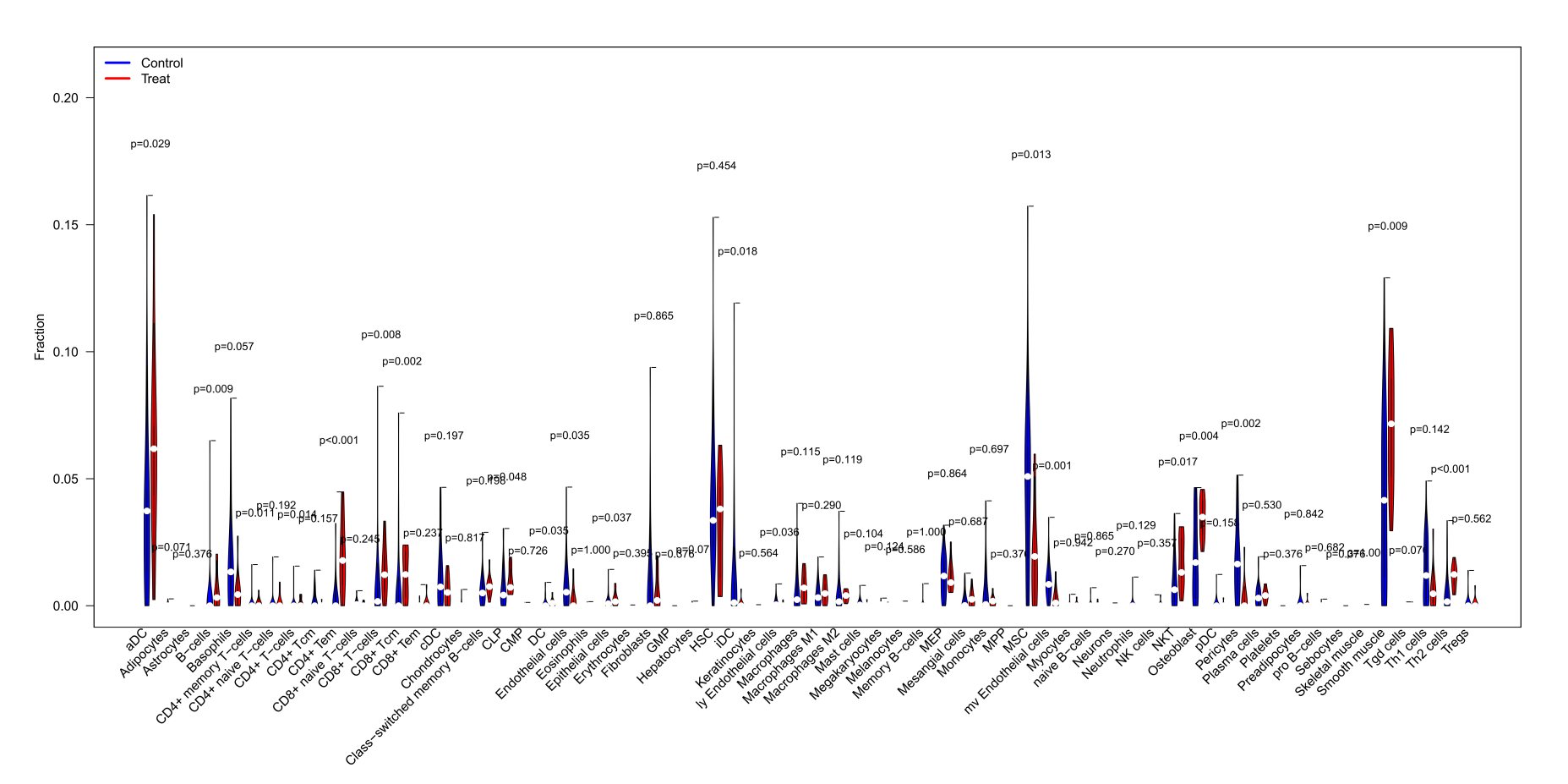


**Fig. S27. Violin plot showing differences in immune cell infiltration levels between AIH and control groups by xCell immune infiltration analysis**


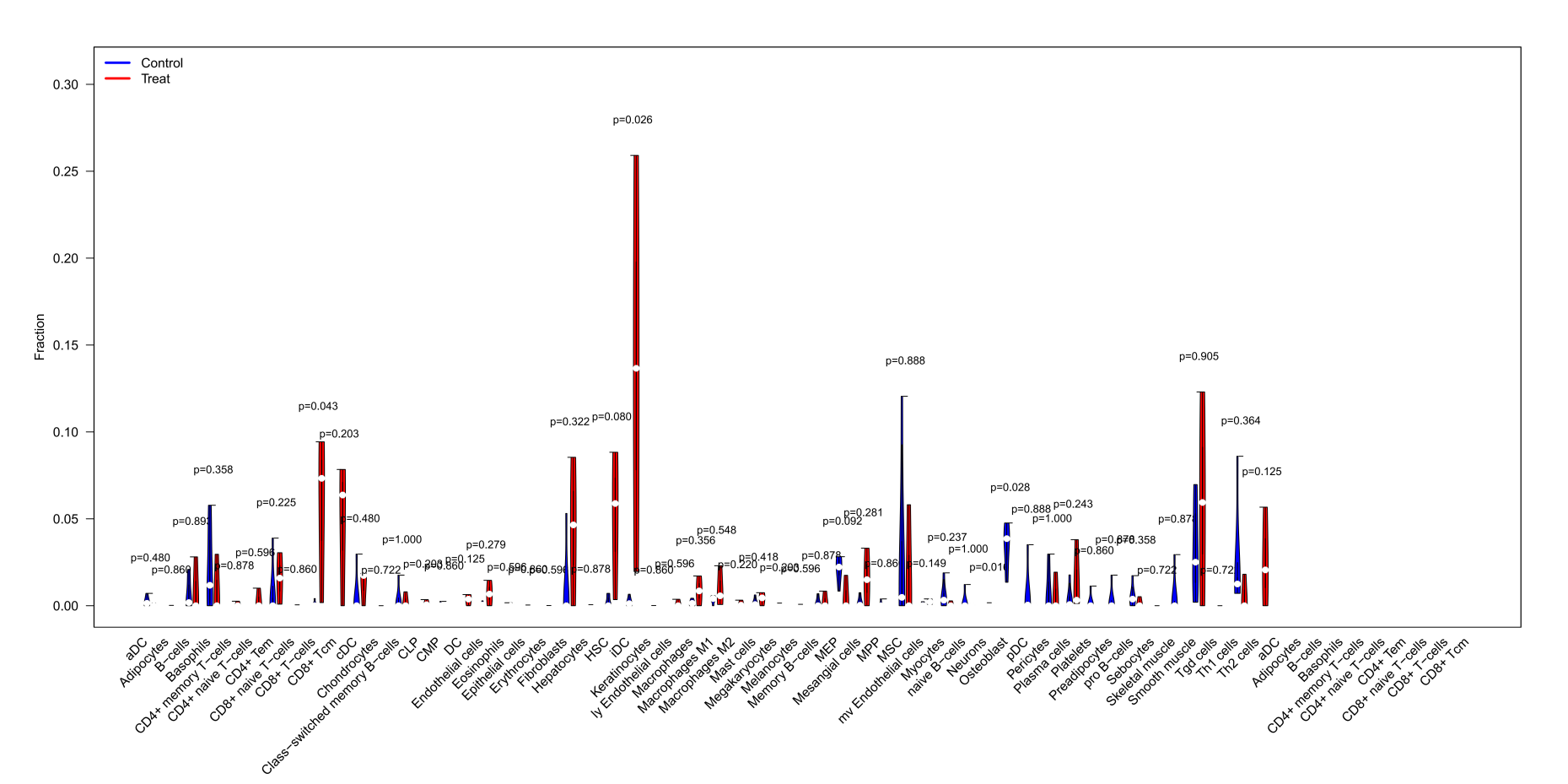


**Fig. S28. Violin plot showing differences in immune cell infiltration levels between PBC and control groups by xCell immune infiltration analysis**


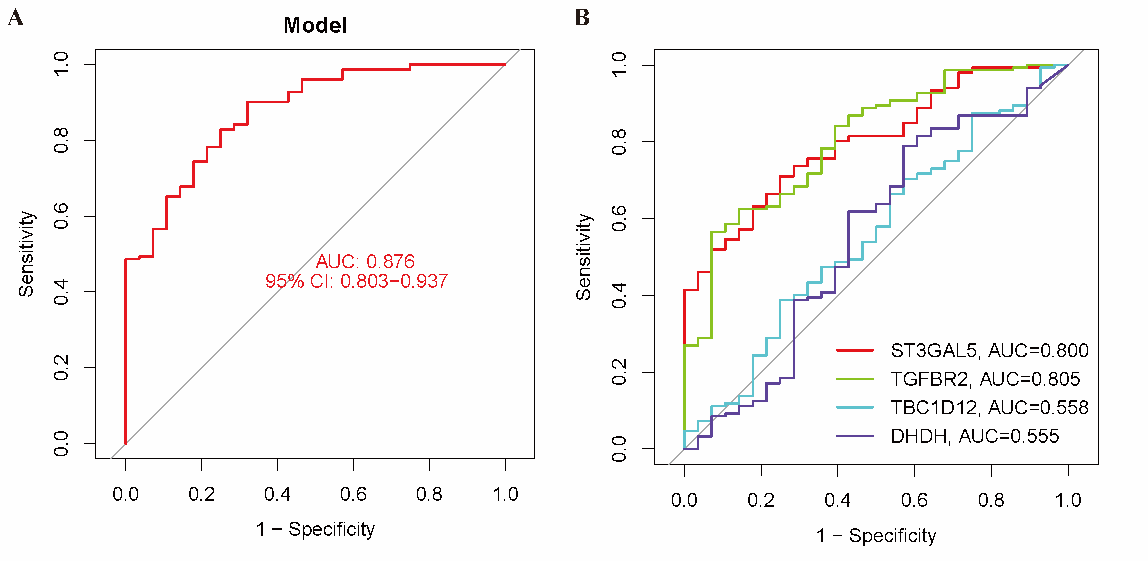


**Fig. S29. Funnel plot for the effect of primary sclerosing cholangitis on anti-EBV ZEBRA antibodies.**  (A) ROC curve for the combined dataset of four genes.(B) ROC curves for each of the four related genes.
